# Promiscuous CYP87A enzyme activity initiates cardenolide biosynthesis in plants

**DOI:** 10.1038/s41477-023-01515-9

**Published:** 2023-09-18

**Authors:** Maritta Kunert, Chloe Langley, Rosalind Lucier, Kerstin Ploss, Carlos E. Rodríguez López, Delia A. Serna Guerrero, Eva Rothe, Sarah E. O’Connor, Prashant D. Sonawane

**Affiliations:** Department of Natural Product Biosynthesis, Max Planck Institute for Chemical Ecology, Jena, Germany

**Keywords:** Secondary metabolism, Molecular engineering in plants, Plant molecular biology, Genetic engineering

## Abstract

Cardenolides are specialized, steroidal metabolites produced in a wide array of plant families^1,2^. Cardenolides play protective roles in plants, but these molecules, including digoxin from foxglove (*Digitalis* spp.), are better known for treatment of congenital heart failure, atrial arrhythmia, various cancers and other chronic diseases^3–9^. However, it is still unknown how plants synthesize ‘high-value’, complex cardenolide structures from, presumably, a sterol precursor. Here we identify two cytochrome P450, family 87, subfamily A (CYP87A) enzymes that act on both cholesterol and phytosterols (campesterol and β-sitosterol) to form pregnenolone, the first committed step in cardenolide biosynthesis in the two phylogenetically distant plants *Digitalis purpurea* and *Calotropis procera*. *Arabidopsis* plants overexpressing these CYP87A enzymes ectopically accumulated pregnenolone, whereas silencing of *CYP87A* in *D. purpurea* leaves by RNA interference resulted in substantial reduction of pregnenolone and cardenolides. Our work uncovers the key entry point to the cardenolide pathway, and expands the toolbox for sustainable production of high-value plant steroids via synthetic biology.

## Abstract (web summary)

Cardenolides such as digoxin from foxglove (*Digitalis* spp.) have been used to treat congenital heart conditions, cancer and other chronic diseases for decades. Here, authors report CYP87A enzymes that catalyse pregnenolone formation, the key entry point to the cardenolide pathway in plants.

## Main

**Fig. 1:**
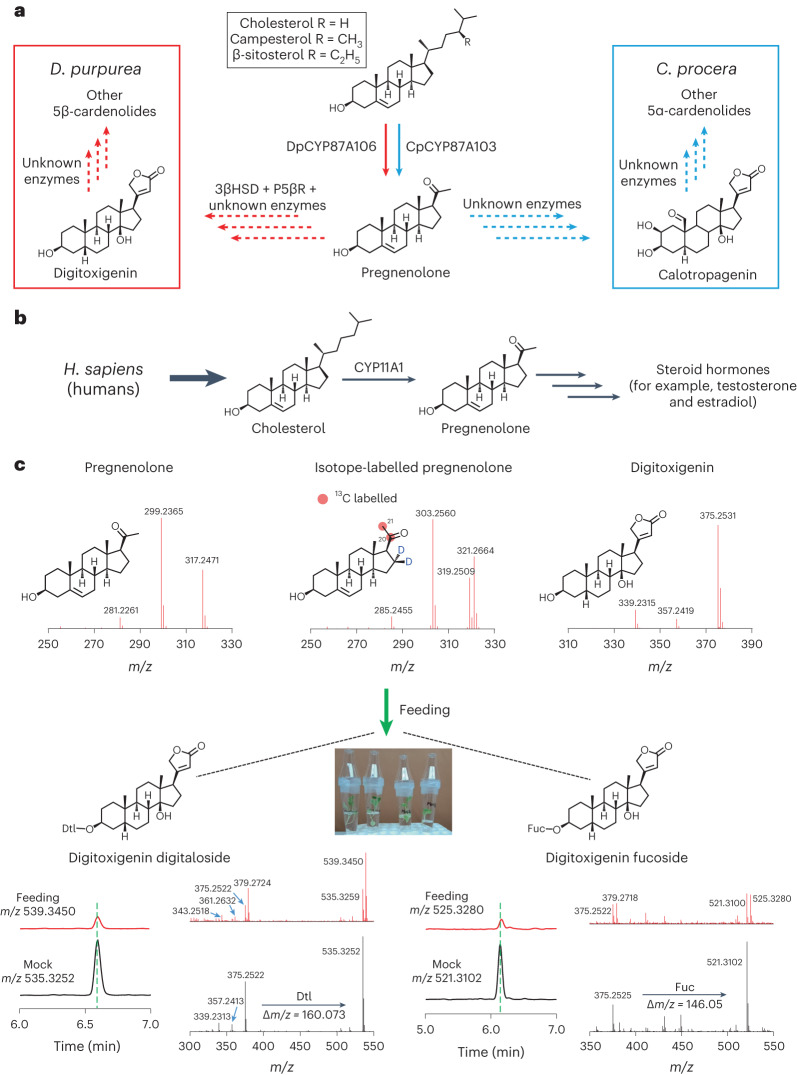
Summary of cardenolide biosynthesis and transformation of pregnenolone to cardenolides by stable isotope-labelled feeding studies. **a**, The proposed biosynthetic pathway for cardenolides, starting from an unconfirmed sterol precursor in *D. purpurea* (red) and *C. procera* (blue). A simplified pathway scheme is presented. Dashed arrows indicate uncharacterized steps in the pathway. DpCYP87A106 and CpCYP87A103 enzymatic steps shown in this study are represented by solid arrows. See Supplementary Fig. 1 for more details of the proposed biosynthetic pathway. **b**, CYP11A1 catalyses the conversion of cholesterol to pregnenolone, a precursor of all other steroid hormones in humans. **c**, LC–MS analysis of cardenolides after feeding of isotope-labelled pregnenolone to 4-week-old *D. purpurea* grown in vitro. Mass fragmentation spectra [M + H]^+^ for authentic pregnenolone, isotope-labelled pregnenolone and digitoxigenin standards are shown at the top. Comparison of extracted ion chromatograms and corresponding tandem mass spectrometry fragmentation spectra of two cardenolides, digitoxigenin digitaloside (left) and digitoxigenin fucoside (right) in feeding (treated with isotope-labelled pregnenolone; red) and mock (treated with de-ionized water; black) samples, respectively. Exogenously fed isotope-labelled pregnenolone was successfully incorporated into cardenolides in *D. purpurea*. *m/z* ions are indicated for each cardenolide under feeding and mock treatments. Dtl, digitalose; Fuc, fucose.

Members of the Apocynaceae (for example, *Calotropis procera*, *Nerium oleander*, *Strophanthus gratus* and *Strophanthus kombe*), Plantaginaceae (for example, *Digitalis lanata* and *Digitalis purpurea*) and Brassicaceae (for example, *Erysimum cheiranthoides*) families produce substantial levels of cardenolides, also known as cardiac glycosides^1,10–13^. The renowned cardenolide example is digoxin, a cardiotonic drug recommended as an essential medicine by the World Health Organization. The use of cardenolide-containing *Digitalis* (foxglove) extracts for the treatment of congestive heart diseases was first reported in 1785^14^. However, only a partial biosynthetic pathway of cardenolides has been proposed, which was based on feeding studies of radioisotope-labelled precursors in *D. lanata* and *D. purpurea* performed in the 1960s^15–18^. These findings demonstrated that cardenolide biosynthesis proceeds through pregnane derivatives (pregnenolone or progesterone) that are probably derived from either cholesterol or phytosterols (for example, β-sitosterol). In contrast, a preferred route for cardenolide biosynthesis involving phytosterols (24-alkyl sterols), not cholesterol, was suggested because of feeding studies in *D. lanata* using inhibitors of phytosterol biosynthesis^19^. Therefore, the specific sterol precursor(s) involvement in cardenolide biosynthesis remains unconfirmed. Despite decades of extensive research on biosynthesis of sterol-derived cardenolides in *Digitalis* spp.^20,21^, only two biosynthetic enzymes, 3β-hydroxysteroid dehydrogenase (3βHSD) and progesterone 5β-reductase (P5βR), have been identified and partially characterized from *Digitalis*^22,23^ and *Erysimum*^24,25^ species. The biosynthetic steps starting from the initial sterol precursor to pregnenolone, and those steps downstream of pregnanolone towards cardiac aglycones (for example, digitoxigenin, calotropagenin) and their corresponding glycosides (for example, digitoxin), remain unidentified in any cardenolide-producing plant species (Fig. 1a; detailed pathway shown for *D. purpurea* in Supplementary Fig. 1).

Formation of pregnenolone from the hypothesized sterol precursor is the first proposed step in the cardenolide biosynthetic pathway in plants (Fig. 1a). In mammals, pregnenolone is formed from cholesterol through the action of cytochrome P450 (CYP), family 11, subfamily A, member 1 (CYP11A1) (Fig. 1b), also known as cholesterol side-chain cleavage enzyme, which catalyses a three-step reaction during steroid hormone biosynthesis (Supplementary Fig. 2). CYP11A1 is a multifunctional mitochondrial enzyme that catalyses two hydroxylation (C22 and C20) reactions followed by a C20–C22 bond cleavage^26^ (Supplementary Fig. 2). In plants, sterol side-chain cleavage enzyme activity on different sterol substrates was evidenced decades ago^27,28^. Although no such plant enzyme has been identified and characterized unambiguously, sterol C22 hydroxylation and other C–C bond cleavage activities are known to be catalysed by diverse CYP classes in the plant kingdom^29–32^. Moreover, previous work showed that nearly all enzymes involved in cholesterol and phytosterol biosynthesis in plants are localized to the endoplasmic reticulum (ER) membrane^33^. Therefore, we speculated that the sterol pool used for cardenolide biosynthesis would be available in the ER membrane, and thus pregnenolone biosynthesis would probably be carried out by a single ER-localized CYP enzyme (as in mammals) or by a set of two or three CYP enzymes.

Before searching for CYP genes, we first wanted to validate that pregnenolone is in fact the precursor for cardenolide biosynthesis in *D. purpurea* because previous feeding studies of isotope-labelled pregnenolone and derivatives showing incorporation into cardenolides were performed in *D. lanata*^15,17,34^, a related *Digitalis* species. We fed stable isotope-labelled pregnenolone ([20,21-^13^C_2_][16,16-D_2_]pregnenolone) to the cut stems of 4-week-old *D. purpurea*. After 2–3 weeks, cardenolides were extracted from collected leaves and analysed using liquid chromatography–mass spectrometry (LC–MS). Labelled digitoxigenin glycosides (digitoxigenin digitaloside and digitoxigenin fucoside) and gitaloxigenin glycoside (verodoxin) were detected by monitoring the [M + H]^+^ ions with mass to charge ratio (*m/z*) values of 539.3450, 525.3280 and 582.3279. For plants treated only with water (mock), the corresponding non-labelled cardenolides were detected by monitoring the [M + H]^+^ ions with *m/z* values of 535.3252, 521.3102 and 579.3154 (Fig. 1c and Supplementary Fig. 3a). These results suggest that both carbons (C20 and C21) and hydrogens (16α and 16β) of pregnenolone were retained during biosynthesis of digitoxigenin-derived cardenolides (that is, digitoxigenin digitaloside and fucoside). In verodoxin, a derivative of gitaloxigenin, it appeared that one hydrogen (16β) from pregnenolone was eliminated during the biosynthesis, which is in agreement with the gitaloxigenin structure (16β-hydrogen modified to 16R; R = OCHO; Supplementary Fig. 3b). As previously observed in *D. lanata*^15,17,34^, our findings confirm that pregnenolone is the central, committed precursor in cardenolide biosynthesis in *D. purpurea*, regardless of the unconfirmed starting sterol substrate.

To identify candidate CYP gene(s) involved in pregnenolone biosynthesis, we selected two cardenolide producers belonging to two distantly related plant families, *D. purpurea* (family Plantaginaceae) and *C. procera* (family Apocynaceae). We reasoned that comparative profiling of cardenolides in different tissues of *D. purpurea* and *C. procera*, coupled with transcriptome datasets from the same tissues, would facilitate gene discovery in these plants. Using ultra-high-performance liquid chromatography coupled to quadrupole time-of-flight mass spectrometry, we examined the cardenolide content in multiple tissue types of *D. purpurea* (young leaves, old leaves, leaf base (winged petiole) and roots) and *C. procera* (young leaves, old leaves, stem and roots). We observed increased accumulation of digitoxin and digitoxigenin fucoside in young and old leaves compared with leaf base and roots of *D. purpurea* (Supplementary Fig. 4a). Although cardenolides were found in all analysed tissue types in *C. procera*, they primarily accumulated in the young leaves (Supplementary Fig. 4b). Finally, we generated corresponding tissue-specific RNA sequencing (RNA-seq) datasets from *D. purpurea* and *C. procera* plants (5–7 weeks old).

**Fig. 2:**
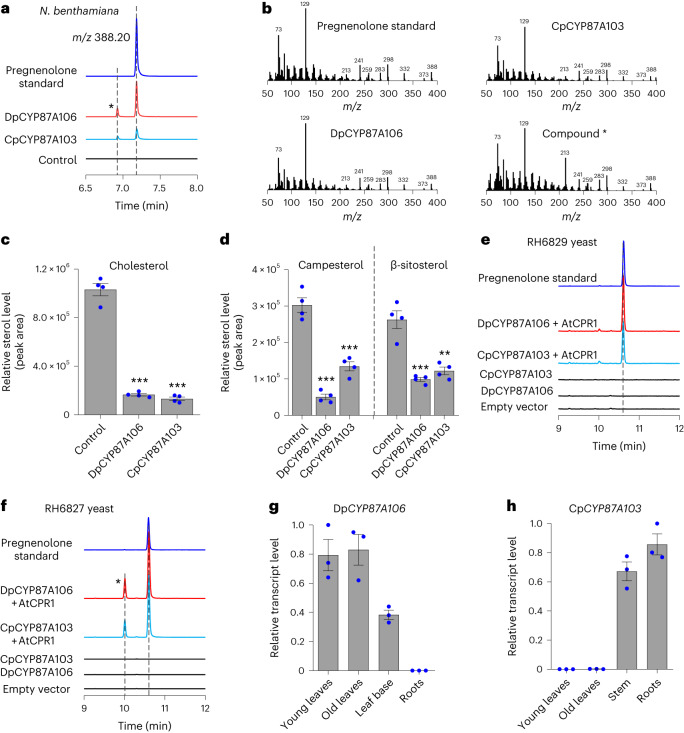
Discovery of DpCYP87A106 and CpCYP87A103 enzymes catalysing pregnenolone biosynthesis in cardenolide-producing plants. **a**, Aligned extracted ion chromatograms presenting the accumulation of pregnenolone (*m/z* 388.20, trimethylsilylated) following transient expression of DpCYP87A106 (red) or CpCYP87A103 (turquoise blue) in *N. benthamiana* compared with an authentic standard (dark blue). The asterisk shows the presence of a new compound at a different retention time, with the same mass spectrum as that of pregnenolone (see **b**). **b**, GC–MS spectra of pregnenolone and the new compound (indicated by the asterisk in **a**) produced in *N. benthamiana* transient experiments compared with pregnenolone standard. **c**,**d**, Levels of cholesterol (**c**) and phytosterols (campesterol and β-sitosterol) (**d**) in *N. benthamiana* leaves that transiently express DpCYP87A106 or CpCYP87A103 compared with control. Leaves infiltrated with empty vector were used as control (in **a**, **c** and **d**). The values in **c** and **d** indicate the mean ± s.e.m. of four biological replicates (*n* = 4) obtained from four independently infiltrated plants. The asterisks indicate significant changes compared with control samples, as calculated by two-tailed Student’s *t*-test; *P < 0.05, **P < 0.01, ***P < 0.001. **e**,**f**, Expression of DpCYP87A106 and CpCYP87A103 in the yeast strains RH6829 (**e**) and RH6827 (**f**), which were engineered to accumulate cholesterol and campesterol, respectively^35^. Co-expression of DpCYP87A106 (red) or CpCYP87A103 (turquoise blue) together with AtCPR1 resulted in pregnenolone formation in RH6829 (**e**) and RH6827 (**f**) yeast strains, respectively. Aligned extracted ion chromatograms for pregnenolone (*m/z* 388.20, trimethylsilylated) are presented. Black represents yeast controls (empty vector (pESC-HIS), DpCYP87A106 alone and CpCYP87A103 alone); blue represents pregnenolone authentic standard. As seen in *N. benthamiana* transient assays (in **a** and **b**), we also detected the compound (shown as asterisk) with the same mass spectrum as that of pregnenolone, but with a different retention time, in the RH6827 yeast experiments. GC–MS was used for sterol profiling. **g**,**h**, Dp*CYP87A106* (**g**) and Cp*CYP87A103* (**h**) gene expression in selected tissues of *D. purpurea* (**g**) and *C. procera* (**h**), as determined by qRT-PCR. The values in **g** and **h** indicate the mean ± s.e.m. of biological replicates obtained from three independent plants (*n* = 3). Source data

We initially selected 13 candidate CYP genes from the *D. purpurea* transcriptome that were highly expressed in young and old leaves compared with roots (Supplementary Fig. 5, left). Each CYP candidate was cloned and expressed in *Nicotiana benthamiana* leaves using an *Agrobacterium tumefaciens*-mediated transient expression system. *N. benthamiana* produces a diverse array of sterols (cholesterol, β-sitosterol, stigmasterol, campesterol and isofucosterol) in substantial amounts^33^; therefore, this host does not require administration of exogenous sterol substrate, making it a highly efficient platform for rapid screening of candidate enzymes in pregnenolone biosynthesis. Metabolic profiling of the leaf extracts by gas chromatography–MS (GC–MS) showed that transient expression of one of these candidates (*D. purpurea* CYP family 87, subfamily A, member 106; DpCYP87A106) led to the production of pregnenolone (Fig. 2a,b), along with substantially decreased levels of three sterols, namely, cholesterol, campesterol and β-sitosterol (Fig. 2c,d). Another compound with the same mass spectrum as that of pregnenolone, but with a different retention time (shown as * in Fig. 2a,b) was also observed but in quantities too low for characterization.

Next we identified five candidate CYP genes in the *C. procera* transcriptome based on their increased expression in young leaves (Supplementary Fig. 5, right), the tissue with the highest accumulation of cardenolide (Supplementary Fig. 4b). However, none of these candidates produced pregnenolone when expressed transiently in *N. benthamiana* and, notably, this candidate list did not contain any member of the CYP87 family. As a next step, we searched for homologues of the *D. purpurea* gene by performing a BLAST search using Dp*CYP87A106* as the query against the *C. procera* transcriptome. A single hit (*C. procera* CYP family 87, subfamily A, member 103; CpCYP87A103) with 54% amino acid sequence identity to DpCYP87A106 was identified. The expression pattern of this gene was poorly correlated with cardenolide presence as it was highly expressed in the stem and roots but had negligible expression in young and old leaves (Supplementary Fig. 5, right). However, when CpCYP87A103 was transiently expressed in *N. benthamiana* leaves, GC–MS analysis of the leaf extract clearly showed the formation of pregnenolone, along with concomitant reduction in cholesterol, campesterol and β-sitosterol (Fig. 2a–d). Therefore, both DpCYP87A106 and CpCYP87A103 enzymes are capable of catalysing pregnenolone formation from an array of sterols. Moreover, cholesterol, campesterol and β-sitosterol appear to be capable of serving as sterol precursors for pregnenolone biosynthesis in *N. benthamiana*. To further support the pregnenolone-forming activity of DpCYP87A106 and CpCYP87A103 enzymes from multiple sterol substrates, we also expressed these enzymes in *Saccharomyces cerevisiae*. As yeast do not produce cholesterol or phytosterols (for example, campesterol), we used previously reported stable yeast strains, RH6829 and RH6827, which were engineered to produce cholesterol and campesterol, respectively^35^. Expression of either DpCYP87A106 or CpCYP87A103 together with the *Arabidopsis* CYP reductase 1 (AtCPR1) in cholesterol-producing RH6829 yeast resulted in formation of pregnenolone (Fig. 2e). Similarly, RH6827 yeast (campesterol generating) cells expressing individual CYP87A enzymes together with AtCPR1 were also able to produce pregnenolone (Fig. 2f). Therefore, both DpCYP87A106 and CpCYP87A103 enzymes produce pregnenolone from either cholesterol or campesterol and, importantly, are sufficient for pregnenolone biosynthesis from sterol precursors in heterologous hosts.

Although cardenolides are found in the leaves of both *D. purpurea* (Supplementary Fig. 4a) and *C. procera* (Supplementary Fig. 4b), the corresponding transcriptome datasets (one replicate per tissue) indicate that Dp*CYP87A106* is predominantly expressed in young and old leaves, whereas Cp*CYP87A103* is mainly expressed in stem and roots (Supplementary Fig. 5). This expression pattern was further validated by quantitative real-time PCR (qRT-PCR) measurements performed in an independent experiment (three biological replicates per tissue, *n* = 3; Fig. 2g,h). Therefore, stem and root tissues appear to be the primary site of pregnenolone biosynthesis in the early cardenolide pathway in *C. procera*, and either pregnenolone or downstream intermediates are probably transported to young and old leaves for further biosynthetic steps. In contrast, in *D. purpurea*, the pregnenolone biosynthetic enzyme and the cardenolides are both located in the leaf, suggesting that intermediates are not transported for cardenolide biosynthesis in this plant. Moreover, the accumulation of cholesterol and phytosterols (for example, campesterol and β-sitosterol) in these tissues of *C. procera* and *D. purpurea* indicate that the starting sterol precursors required for pregnenolone biosynthesis are available in these plants (Supplementary Fig. 6).

**Fig. 3:**
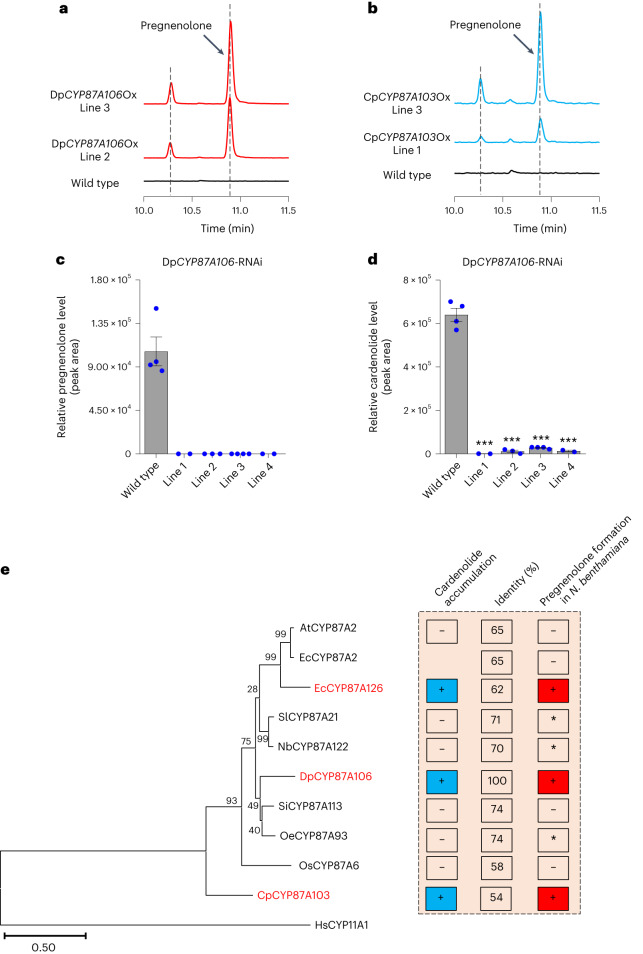
Silencing of Dp*CYP87A106* by RNAi in *D. purpurea* confirms its role in cardenolide biosynthesis. **a**,**b**, De novo production of pregnenolone in transgenic *Arabidopsis* leaves overexpressing Dp*CYP87A106* (red) (**a**) or Cp*CYP87A103* (blue) (**b**) compared with wild-type leaves (black). Lines 2 and 3 are independent Dp*CYP87A106*-Ox transgenic *Arabidopsis* lines, whereas lines 1 and 3 are independent Cp*CYP87A103*-Ox transgenic *Arabidopsis* lines. Extracted ion chromatograms are shown. GC–MS was used for sterol profiling. **c**, Levels of pregnenolone in leaves of Dp*CYP87A106*-RNAi transgenic *D. purpurea* lines compared with wild type. Pregnenolone was not detected in the Dp*CYP87A106*-RNAi lines. **d**, Knockdown of Dp*CYP87A106* resulted in almost complete loss of cardenolides in transgenic *D. purpurea* leaves. The relative cardenolide levels shown are the sum of the peak areas obtained from six cardenolides (verdoxin, digitoxigenin fucoside, digitoxin, purpurea glycoside A, purpurea glycoside B and glucogitaloxin) that typically accumulate in *D. purpurea*^10^. Four independent Dp*CYP87A106*-RNAi transgenic *D. purpurea* lines (lines 1, 2, 3 and 4) were generated using a stable transformation approach and analysed in the T_0_ generation. Values in **c** and **d** indicate means ± s.e.m. of biological replicates (*n* = 4 for wild type and *n* ≥ 2 for individual transgenic lines). Biological replicates (for example, for line 1, *n* = 2) refer to the individual plants that emerged from the same callus explant. Asterisks indicate significant changes compared with wild-type samples, as calculated by two-tailed Student’s *t*-test; *P < 0.05, **P < 0.01, ***P < 0.001. **e**, Phylogenetic context of CYP87A proteins obtained from cardenolide-producing and cardenolide-free plants. The percentage identity at the amino acid level for each CYP87A protein compared with DpCYP87A106 is presented. The blue squares (with the + sign) indicate presence of chemotype (that is, cardenolide) in a particular plant species. In planta pregnenolone-producing activity of CYP87A clade proteins seen in *N. benthamiana* transient expression experiments is shown by red squares (with the + sign). Increased pregnenolone accumulation by three CYP87A proteins (marked in red), EcCYP87A126, DpCYP87A106 and CpCYP87A103, correlated well with the presence of cardenolides in these corresponding plant species. The asterisk represents negligible pregnenolone-formation activity in transient experiments by CYP87A enzymes (for example, tomato) from non-cardenolide-producing plants. CYP87A sequences from the following species were used in phylogenetic analysis: *S. lycopersicum* (Sl; tomato), *N. benthamiana* (Nb), *O. sativa* (Os; rice), *A. thaliana*, *S. indicum* (Si; sesame), *O. europaea* (Oe; olive), *E. cheiranthoides*, *D. purpurea*, *C. procera* and *H. sapiens* (humans). The amino acid sequences used in the phylogenetic analysis are provided in Supplementary Data 1. Percentage bootstrap values are shown at the nodes of each branch. Scale bar represents branch lengths measured in the number of amino acid substitutions per site. Source data

We next assessed whether DpCYP87A106 and CpCYP87A103 could produce pregnenolone in a non-cardenolide-producing model plant such as *Arabidopsis thaliana*. We generated stable transgenic *A. thaliana* lines overexpressing either Dp*CYP87A106* (named Dp*CYP87A106-*Ox) or Cp*CYP87A103* (named Cp*CYP87A103-*Ox). We observed de novo production of pregnenolone in the leaves of Dp*CYP87A106-*Ox and Cp*CYP87A103-*Ox plants (Fig. 3a,b), confirming that both CYP87A subfamily enzymes catalyse formation of pregnenolone in a plant unrelated to *N. benthamiana*.

To definitively establish whether these enzymes play a direct role in cardenolide biosynthesis, we used RNA-mediated interference (RNAi) to silence Dp*CYP87A106* (Dp*CYP87A106*-RNAi) in *D. purpurea*. Dp*CYP87A106* transcript levels were significantly reduced in the leaves of Dp*CYP87A106*-RNAi plants (Supplementary Fig. 7a). Furthermore, we could not detect pregnenolone in the leaves (6–8 weeks old) of Dp*CYP87A106*-RNAi lines (Fig. 3c). Dp*CYP87A106*-RNAi leaves also showed a substantial decrease in cardenolide levels compared with wild type *D. purpurea* leaves (Fig. 3d). Analysis of sterols in Dp*CYP87A106*-RNAi leaves showed a significant increase in campesterol levels, with no major change in cholesterol (Supplementary Fig. 7b), suggesting that campesterol could be the preferred sterol precursor for pregnenolone formation in *D. purpurea*. To examine whether the low cardenolide chemotype in Dp*CYP87A106*-silenced lines could be rescued, we fed the pregnenolone precursor to cut leaf discs of Dp*CYP87A106*-RNAi lines. After 4–6 days, leaf discs were extracted and analysed for cardenolides using LC–MS. The cardenolide content in pregnenolone-treated Dp*CYP87A106*-RNAi lines remain unaffected compared with that of control Dp*CYP87A106*-RNAi lines (water treated). However, pregnenolone-treated Dp*CYP87A106*-RNAi plants (lines 1 and 2) showed accumulation of 5β-pregnane-3,20-dione and 5β-pregnane-3β-ol-20-one, which are known downstream intermediates of the cardenolide pathway (Supplementary Fig. 8). We did not detect these pathway intermediates in the control Dp*CYP87A106*-RNAi (water treated) plants. As the cut leaf discs are only metabolically active for less than a week, we hypothesize that this was not enough time for all the downstream enzymatic steps required for complete rescue of cardenolide biosynthesis. Unfortunately, these feeding studies were not technically possible with the mature transformed *Digitalis* plants (9–10 months old).

The cholesterol side-chain cleavage enzyme in humans, *Homo sapiens* CYP11A1 (HsCYP11A1), catalyses a well-studied, three-step reaction sequence in which two stereoselective successive hydroxylations of cholesterol (at C20 and C22), followed by C20–C22 cleavage of 20,22-dihydroxycholesterol, results in the formation of pregnenolone^36^. To compare the potential mechanism of the plant enzyme with the human one, we generated structural models of DpCYP87A106 and CpCYP87A103 (ref. ^37^). The catalytic pockets of DpCYP87A106 and CpCYP87A103 (Supplementary Fig. 9, shown in blue and green, respectively) were highly conserved despite the overall low homology of the two proteins (54% amino acid identity). Next we performed docking of cholesterol, 22-hydroxycholesterol and 20,22-dihydroxycholesterol substrates in the active site of DpCYP87A106 (Supplementary Fig. 10a,c,e) and CpCYP87A103 (Supplementary Fig. 10b,d,f). The large number of non-polar residues within the catalytic pocket suggests steric hindrance is important to correctly orientate the substrate, as reported for HsCYP11A1 (ref. ^36^). For each docked sterol, we could achieve a substrate orientated with the hydroxylation (for cholesterol or 22-hydroxycholesterol) or C–C cleavage position (for 20,22-dihydroxycholesterol) between 2.7 Å and 6.1 Å of the ferric haem, suggesting that a three-step reaction sequence, as proposed for HsCYP11A1, is plausible^36^. Similar results were achieved when DpCYP87A106 and CpCYP87A103 homology models were docked with campesterol, 22-hydroxycampesterol and 20,22-dihydroxycampesterol (Supplementary Fig. 11a–f). From the substrate-docking studies, we identified Ile210 in DpCYP87A106 (Ile209 in CpCYP87A103) as a residue that might control substrate orientation by steric hindrance (Supplementary Fig. 12a,b). Site-directed mutagenesis of DpCYP87A106 Ile210 and CpCYP87A103 Ile209 to either phenylalanine or tryptophan led to enzyme mutants that produced negligible or no pregnenolone when expressed transiently in *N. benthamiana* (Supplementary Fig. 12c,e). Both point mutations resulted in restoration of the levels of cholesterol and the phytosterols campesterol and β-sitosterol to those observed in control plants (Supplementary Fig. 12d,f), suggesting that Ile209 or Ile210 is important for sterol substrate binding in pregnenolone formation.

To explore the evolutionary aspect of the CYP87A family enzymes involved in cardenolide biosynthesis, we conducted a BLAST search of DpCYP87A106 against National Center for Biotechnology Information (NCBI) and public databases and extracted the putative orthologous coding sequences from an additional cardenolide-producing plant (*E. cheiranthoides*), and several cardenolide-free plants (*A. thaliana*, *Oryza sativa* (rice), *Solanum lycopersicum* (tomato), *Sesamum indicum* (sesame), *Olea europaea* (olive) and *N. benthamiana*). These identified CYP87A proteins share 54–74% homology with DpCYP87A106, and their existence in both cardenolide-free and cardenolide-producing species suggests that CYP87A is widely conserved in plants (Fig. 3e). However, so far no CYP87A subfamily member had been functionally characterized. We identified two CYP87A proteins (EcCYP87A2 and EcCYP87A126) sharing 75% amino acid identity in *E. cheiranthoides*, a substantial cardenolide producer from Brassicaceae family. We assayed each of these CYP87A proteins (10 in total including DpCYP87A106 and CpCYP87A103; Fig. 3e and Supplementary Fig. 13) for pregnenolone-forming activity via transient expression in *N. benthamiana*. In addition to DpCYP87A106 and CpCYP87A103, transient expression of EcCYP87A126 led to increased accumulation of pregnenolone (Supplementary Fig. 13), suggesting its involvement in *Erysimum* cardenolide biosynthesis. In contrast, we detected only negligible levels of pregnenolone with CYP87A members from tomato, olive and *N. benthamiana* (Supplementary Fig. 13). Phylogenetic analysis shows a separate small clade containing *A. thaliana* CYP87A2 (AtCYP87A2) and EcCYP87A2 enzymes, both of which lack pregnenolone formation activity (Fig. 3e). Clear separation of EcCYP87A126 from EcCYP87A2 and the *Arabidopsis* enzyme (AtCYP87A2) suggest that EcCYP87A126 may have arisen via gene duplication from an EcCYP87A2-like ancestor before undergoing neo-functionalization to acquire pregnenolone-formation activity (Fig. 3e). However, we observed no such clades for *Digitalis* and *Calotropis* enzymes. Thus, the evolutionary mechanism by which DpCYP87A106 and CpCYP87A103 acquired the catalytic activity required for pregnenolone biosynthesis remains undetermined.

CYP87D subfamily members, such as CYP87D16 (*Maesa lanceolata*), CYP87D20 (*Cucumis sativus*) and CYP87D18 (*Siraitia grosvenorii*), catalyse single or multiple hydroxylation and/or oxidation reactions in triterpene biosynthesis^38^. Our study shows that the CYP87A subfamily members DpCYP87A106 from *D. purpurea* and CpCYP87A103 from *C. procera* act on sterol molecules in cardenolide biosynthesis. Characterization of CYP87A activity in pregnenolone formation is a significant step towards resolving one of the most complex and lengthy biosynthetic pathways of sterol-derived cardenolides in distantly related plant families (for example, Apocynaceae (*Calotropis*, *Nerium*, *Asclepias*), Plantaginaceae (*D. lanata*), Asparagaceae (*Convallaria majalis*) and Moraceae (*Antiaris toxicaria*)). Furthermore, pregnenolone serves as a precursor for many high-value steroidal molecules, including hormones (for example, progesterone, testosterone, androsterone, estradiol and many more) and drugs (for example, corticosteroids and anti-inflammatory and anti-allergic medications). Discovery of the CYP87A enzymes provides a crucial tool for building a platform for engineering the sustainable production of high-value steroids using state-of-the-art synthetic biology applications.

## Methods

### Plant material

*D. purpurea* (foxglove) and *C. procera* plants were grown in a climate-controlled greenhouse at 24 °C during the day and 18 °C during the night, with natural light. *N. benthamiana* plants were grown in a growth room maintained at 23 ± 2 °C with a 16 h day and 8 h night regime.

### Analytical standards

Digitoxigenin and digitoxin standards (Sigma-Aldrich) were dissolved in methanol at a concentration of 1 mg ml^−1^. Sterol standards, cholesterol, campesterol, β-sitosterol, pregnenolone and [20,21-^13^C_2_][16,16-D_2_]pregnenolone were purchased from Sigma-Aldrich and, unless stated otherwise, were dissolved in ethanol to a concentration of 1 mg ml^−1^. 5β-Pregnane-3,20-dione and 5β-pregnane-3β-ol-20-one (pregnanolone) standards were purchased from Steraloids and dissolved in ethanol to a concentration of 1 mg ml^−1^ for further use.

### Plant extract preparation and LC–MS-based targeted profiling of cardenolides

Preparation of extracts of *D. purpurea* (young leaves, old leaves, leaf base (winged petiole) and roots) and *C. procera* (young leaves, old leaves, stem and roots) tissues (*n* = 3) was performed as previously described^10,11^. Briefly, selected plant tissues were frozen in liquid nitrogen and ground to a fine powder using a TissueLyser II (Qiagen) homogenizer or mortar and pestle. Frozen tissue (100 mg) was extracted with 80% methanol, briefly vortexed for 2 min and then incubated at 65 °C for 12 min. Finally, the extracts were centrifuged for 15 min at 20,000*g* and filtered through 0.22 μm filters. Samples were analysed using a Thermo Scientific UltiMate 3000 ultra-high-performance liquid chromatography system coupled to an Impact II UHR-Q-ToF (ultra-high-resolution quadrupole time of flight) mass spectrometer (Bruker Daltonics) with the standard (22 min, positive mode) run conditions as follows: 5% B for 1 min, 5% B to 95% B in 12 min, 95% B for 2 min, changing to 100% B within 0.2 min and continuing at 100% B for 2.8 min, and finally returning to the initial conditions (5% phase B) within 0.5 min. The column was equilibrated with 5% B for another 3.8 min before next injection.The mobile phase consisted of 0.1% formic acid in water (phase A) and acetonitrile (phase B). Separation of metabolites was performed on a 50 × 2.1 mm, 1.7 µm Acquity UPLC C18 column (Waters). The flow rate was 0.3 ml min^−1^, and the column temperature was kept at 35 °C. Mass spectrometry was performed in positive electrospray ionization mode (capillary voltage 4,000 V; end plate offset 500 V; nebulizer pressure 2.2 bar; drying gas, nitrogen at 250 °C and 10 l min^−^^1^). Mass spectrometry data were recorded at 12 Hz, ranging from 100 *m/z* to 1,200 *m/z* in auto tandem mass spectrometry mode with an active exclusion window of 0.2 min. Fragmentation was triggered on an absolute threshold of 400 counts and restricted to a total cycle time of 0.5 s, with dynamic collision energy (20–50 eV). To calibrate spectrum recording for mass spectrometry, each run was initiated with the direct source infusion of a sodium formate–isopropanol calibration solution (using external syringe pump at 0.18 ml h^−1^). The initial 1 min of the chromatographic gradient was directed towards the waste. Cardenolides were identified by comparing the retention time and mass spectra of authentic standards analysed on the same instrument (see ‘Analytical standards’ above). When the corresponding standards were not available, metabolites were putatively identified by comparing their retention times, elemental composition and mass fragmentation pattern with those described in the literature^10,12,13^. Relative quantification of the cardenolides was performed using Bruker Compass Data Analysis (v.5.3) software.

### Transcriptome analysis

Total RNA from 5–7-week-old *D. purpurea* (young leaves, old leaves, leaf base (winged petiole) and roots) and *C. procera* (young leaves, old leaves, stem and roots) was extracted using the RNeasy Mini Kit (Qiagen), according to the manufacturer’s instructions. High-quality RNA (260 nm:280 nm ratio of 2.0–2.2; 260 nm:230 nm ratio of 2.1–2.4) samples were submitted to Novogene (https://en.novogene.com/) for preparing mRNA libraries and further RNA-seq (paired-end 2× 150, ~40M reads per sample) using the company’s standard protocols. The raw sequencing data were assembled using an in-house pipeline developed for transcriptome analysis. In brief, de novo transcriptome assemblies were generated for *D. purpurea* and *C. procera* from cleaned, trimmed reads using Trinity^39^. The transcriptome assemblies were refined using the CD-HIT Suite to group transcripts with greater than 90% identity, and only the longest transcript was retained in each case. Transdecoder (https://github.com/TransDecoder/TransDecoder) was used to identify candidate-coding regions within transcript sequences. Functional annotation was then performed by running BLAST against the UniProt and Pfam database. Finally, gene expression, measured in transcripts per million, was calculated using Salmon^40^.

### Feeding experiment

Isotope-labelled pregnenolone (2 mg) was dissolved in 100 µl ethanol. *D. purpurea* plants grown in vitro were used in feeding experiments. Briefly, 4-week-old plants were trimmed by cutting off the base stem, placed into plastic tubes with de-ionized water (~2 ml) and then fed with stable isotope-labelled pregnenolone (final concentration, 0.23 mM). Plants treated with de-ionized water were used as control (mock). Treated and mock plants were further maintained in a growth room at 25 °C, with a 16:8 hour photoperiod under LED light. After 2–3 weeks, leaves were collected and analysed by LC–MS for the accumulated cardenolides, as described above.

### Generation of Dp*CYP87A106* transgenic *D. purpurea* plants

The Dp*CYP87A106*-silencing construct (Dp*CYP87A106*-RNAi) was generated by introducing a 321 bp Dp*CYP87A106* fragment (forward primer, GCGGCCGCGAGCTGGTATCCAAGCACCTTC; reverse primer, GGCGCGCCGCTGATCAAACCATCTATGAACGCC) to pENTR/D-TOPO (Invitrogen; by NotI and AscI) and then cloning to the pK7GWIWG2 (II) binary vector using the Gateway LR Clonase II enzyme mix (Invitrogen). The vector was stably transformed to into *D. purpurea* as follows: seeds of *D. purpurea* were surface sterilized by dipping in 70% ethanol for 30 s, followed by 15 min in 1% sodium hypochlorite and then rinsing three to five times in sterile distilled water. The sterile seeds were germinated in half-strength MS221 (Murashige and Skoog) medium, supplemented with 1% sucrose and 0.8% plant agar (Duchefa) adjusted to pH 5.8. Plants were maintained in a growth room at 25 °C, with a 16:8 hour photoperiod under LED light. Overnight-grown *A. tumefaciens* (strain GV3101) bacteria containing the binary vector pK7GWIWG2 (II)–Dp*CYP87A106* were centrifuged and the pellet was resuspended (OD_600_ = 0.4) in CT medium containing 3% glucose and 0.2 g l^−1^ KH_2_PO_4_, used for liquid co-cultivation. Well-developed first and second pairs of leaves from 5-week-old *D. purpurea* plants germinated in vitro were used as explants. The distal edges of leaf explants were cut out and briefly dipped in the bacterial suspension (supplemented with 100 μM acetosyringone), incubated in the dark for 20–30 min, placed on CT solid media, already supplemented with 100 μM acetosyringone, 75 µg ml^−1^ DTT, 2 μg ml^−1^ zeatin and 0.2 μg ml^−1^ indole-3-acetic acid (IAA). After 48 h of co-cultivation in dark conditions, explants were transferred to MSI (shoot induction) medium containing zeatin (2 μg ml^−1^), IAA (0.2 μg ml^−1^), kanamycin (50 μg ml^−1^) and ticarcillin (250 μg ml^−1^) for 3 weeks, and then transferred onto MSII (shoot elongation) medium containing zeatin (1 μg ml^−1^), zeatin riboside (1 μg ml^−1^), IAA (0.2 μg ml^−1^), kanamycin (50 μg ml^−1^) and ticarcillin (100 μg ml^−1^). Well-developed shoots (after 3–4 weeks) were excised and transferred to MSIII (rooting medium) containing indole-3-butyric acid (2 μg ml^−1^), kanamycin (50 μg ml^−1^) and ticarcillin (100 μg ml^−1^). Finally, plantlets with roots were transferred to soil for hardening in greenhouse and maintained further. Wild-type (non-transformed) *D. purpurea* plants germinated in vitro were used as a control in transgenic plant analysis. Positive transgenic lines were selected by qRT-PCR and then used for LC–MS and GC–MS-based metabolite analysis. Four independent transgenic lines (lines 1, 2, 3 and 4) were successfully generated after transformation and analysed in the T_0_ generation. Each transgenic plant line was generated from callus (produced from leaf explants) during the plant transformation process. In the case of *D. purpurea*, multiple shoots emerged from a single callus explant in tissue culture, and these shoots became multiple plantlets after passing through the shoot elongation, rooting, acclimatization and hardening steps of plant transformation. Therefore, biological replicates here (for example, for line 1, *n* = 2 biological replicates) refer to the individual plants that each emerged from the same callus explant.

### qRT-PCR

Total RNA was isolated from different tissues of *D. purpurea* and *C. procera* plants and from transgenic *D. purpurea* (leaves) using the RNeasy Mini Kit (Qiagen) according to the manufacturer’s instructions. Unless stated otherwise, at least three biological replicates from each genotype were used for gene expression analysis (*n* ≥ 3) by qRT-PCR. For transgenic *D. purpurea* lines, biological replicates (*n* ≥ 2) were used. DNase I-treated (Sigma-Aldrich) RNA was reverse transcribed using a high-capacity cDNA reverse transcription kit (Applied Biosystems). Gene-specific oligonucleotides were designed with Primer BLAST software (NCBI). *UBIQUITIN10* (ref. ^41^) and *ACTIN*^12^ genes were used as the reference genes for *Digitalis* and *Calotropis*, respectively, in expression analysis.

### Transient expression in *N. benthamiana*

For transient overexpression, all candidate genes, including Dp*CYP87A106* and Cp*CYP87A103* (list provided in Supplementary Fig. 5), were cloned into destination vector 3Ω1 using GoldenBraid cloning^42^ and transformed into *A. tumefaciens* (GV3101) by electroporation. Single clones with each target construct were inoculated into 10 ml of LB medium supplemented with antibiotics (250 μg ml^−1^ spectinomycin and 50 μg ml^−1^ gentamicin) and cultures were grown overnight at 28 °C with shaking (200 rpm). Cells were centrifuged at 2,000*g* for 15 min and the pellet was resuspended in 5 ml of infiltration buffer (50 mM MES buffer (pH 5.6), 10 mM MgCl_2_, 150 μM acetosyringone). After another round of centrifugation, the pellet was resuspended again in 10 ml of infiltration buffer and incubated at room temperature for 2 h. *Agrobacterium* suspensions (OD_600_ = 0.4 for each strain) were infiltrated into 4–6-week-old *N. benthamiana* leaves. After 5 days, leaves were collected for further LC–MS-based cardenolide analysis and GC–MS-based sterol analysis. Biological replicates consisted of several leaves collected from different infiltrated plants. Leaves infiltrated with empty vector were used as controls. In other experiments, first site-directed mutants were generated for DpCYP87A106 (I210F and I210W) and CpCYP87A103 (I209F and I209W) and subsequently cloned into the 3Ω1 vector. The resulting constructs were used for transient expression in *N. benthamiana* as described above.

### GC–MS analysis of sterols

Powdered, frozen tissues (100 mg) were saponified at 70 °C for 2 h in 0.6 ml of 20% KOH (w/v) in 50% ethanol. Samples were mixed every 20 min during this procedure. Upon cooling to room temperature, samples were extracted three times with 0.5 ml hexane. The combined hexane phases were evaporated to dryness using a gentle stream of nitrogen, resuspended in 60 µl of *N*-methyl-*N*-(trimethylsilyl) trifluoroacetamide (MSTFA), and incubated for 10 min at room temperature and then for 20 min at 65 °C. Each sample was transferred to the glass insert, and 1 µl was injected onto GC–MS with or without dilution, depending on the run conditions and analyte concentrations. The GC–MS system comprised a GC PAL autosampler (CTC Analytics), a trace 1310 GC ultra-gas chromatograph equipped with a split–splitless injector and ISQ quadrupole mass spectrometer (Thermo Scientific). GC was performed on two different columns: column 1, 30 m × 0.25 mm × 0.1 μm Zebron ZB-5HT with 5 m guard mass spectrometry column (Phenomenex; for example, data generated using this column was shown in Fig. 2a); column 2, 30 m × 0.25 mm × 0.25 μm Zebron ZB-5 with 10 m guard mass spectrometry column (Phenomenex; generated data shown in Fig. 3a,b). Samples were analysed in the split mode and the inlet temperature was set at 250 °C. Analytes were separated using the following chromatographic conditions: helium was used as carrier gas at a flow rate of 1.1 ml min^−1^. The thermal gradient used for column 1 started at 160 °C (hold 1.5 min), ramped up to 270 °C at 30 °C min^−1^ and then ramped up to 290 °C at 1.5 °C min^−1^ (hold 15 min); the thermal gradient used for column 2 started at 160 °C (hold 1.5 min), ramped up to 270 °C at 30 °C min^−1^, ramped up to 290 °C at 1 °C min^−1^ (hold 10 min) and then finally up to 300 °C at 30 °C min^−1^ (hold 2.5 min). Eluents were fragmented in the electron impact mode with an ionization voltage of 70 eV, and the mass spectrometry transfer line temperature was set at 290 °C and the ion source at 250 °C. The reconstructed ion chromatograms and mass spectra were evaluated using Xcalibur software (v.4.2.47; Thermo Scientific). Sterol compounds were identified by comparing their retention index and mass spectrum with those generated for authentic standards (trimethylsilylated) analysed on the same instrument (see ‘Analytical standards’ above) and those reported in the literature^43–45^.

### Expression of Dp*CYP87A106* and Cp*CYP87A103* genes in yeast and metabolite extraction

Both Dp*CYP87A106* and Cp*CYP87A103* genes were cloned separately into pESC-HIS plasmid using BamHI and XhoI restriction enzymes. The resulting pESC-HIS plasmid harbouring either Dp*CYP87A106* or Cp*CYP87A103* was used as a template further to clone At*CPR1* (AT4G24520) using NotI and SacI restriction enzymes. Final pESC-HIS–Dp*CYP87A106* and pESC-HIS–Cp*CYP87A103* constructs with or without At*CPR1* were transformed into RH6827 and RH6829 yeast strains using the Yeastmaker yeast transformation system (Clontech). Both RH6827 and RH6829 yeast strains were engineered to accumulate campesterol and cholesterol, respectively^35^. Transformed yeast was grown on synthetic drop-out medium supplemented with appropriate amino acids (without histidine) and 2% glucose. Colony PCR was used to confirm the presence of the transgene in transformed yeast strains. Positive yeast clones were grown for 36 h at 30 °C in 2 ml synthetic drop-out liquid medium supplemented with appropriate amino acids (without histidine) and 2% glucose. Then the yeast cell cultures were centrifuged at 700*g* for 10 min, and pellets were resuspended in 10 ml H_2_O and centrifuged again. Finally, washed yeast cells were transferred to synthetic drop-out medium supplemented with appropriate amino acids (without histidine) and 2% galactose, and grown further for 24 h at 30 °C. After induction of gene expression, yeast cells were centrifuged at 700*g* for 10 min, the pellet was resuspended in 0.6 ml of saponification solution (20% KOH (w/v) in 50% ethanol) and transferred to a 2 ml Eppendorf tube. A volume of glass beads (0.5 mm diameter) roughly half of the cell resuspension was added to the Eppendorf tube, and this was vortexed 10 times for 1 min. Between each vortexing cycle, cells were kept on ice for 1 min. Lysed cells were saponified at 70 °C for 2 h, extracted three times with hexane, dried and resuspended in 50 µl of MSTFA. Derivatized samples were incubated for 20 min at 65 °C and analysed on GC–MS for sterol analysis, as described above with one modification in the GC column. For the yeast experiment, GC was performed on the following newly installed column: 30 m × 0.25 mm × 0.25 μm Zebron ZB-5 with 10 m guard mass spectrometry column (Phenomenex; generated data shown in Fig. 2e,f).

### Generation of *Arabidopsis* transgenic lines

*A. tumefaciens* GV3101 strains harbouring the 3Ω1–Dp*CYP87A106* and 3Ω1–Cp*CYP87A103* constructs were transformed separately into *Arabidopsis* (Col-0) plants using the floral dip method as previously described^46^. Leaves from positive transgenic lines were analysed further for pregnenolone formation using GC–MS as described above.

### Phylogenetic analysis

CYP87A clade sequences from various plants were obtained from NCBI and public databases using the BlastP program (using DpCYP87A106 as the query). Sequence alignments were performed using ClustalOmega^47^. The maximum-likelihood tree was inferred in MEGAX^48^ with the following parameters: 1,000 bootstrap replications, Poisson model, discrete gamma distribution (five categories) and partial deletion. Evolutionary distances are in units of number of amino acid substitutions per site. The amino acid sequences used in the phylogenetic analysis are provided in Supplementary Data 1.

### Synthetic gene sequences

De-novo-synthesized *CYP87A* gene sequences were obtained from Twist Bioscience for the following plant species: *O. sativa* (rice), *S. indicum* (sesame), *O. europaea* (olive) and *E. cheiranthoides*. As we could not amplify *CYP87A* from *S. lycopersicum* (tomato) and *N. benthamiana*, synthetic versions of these genes were also acquired from Twist Bioscience. All the synthetic *CYP87A* genes were cloned into 3Ω1 and used for transient expression in *N. benthamiana* as described above. The primers used for cloning are provided in Supplementary Table 1.

### DpCYP87A106 and CpCYP87A103 modelling and docking studies

Homology models of DpCYP87A106 and CpCYP87A103 were generated using ColabFold, accessed via Google Colaboratory, and run using default parameters^37^. The highest-scoring model of each protein, generated based on the predicted local distance difference test, was used for further study. Docking was performed using AutoDock Vina on the Webina webserver with default parameters (exhaustiveness, 8)^49^. Substrates and receptors were prepared for docking using PDBQTConvert. Models with ligand orientations in which the hydroxylation or C–C cleavage site was in proximity to the ferric heme were selected for further study; this orientation was not always the lowest possible energy solution. Results for modelling and docking studies were visualized using PyMol (v2.0; PyMOL Molecular Graphics System; Schrödinger).

### Pregnenolone feeding to Dp*CYP87A106*-RNAi *D. purpurea* lines

Pregnenolone (20 mg) was dissolved in 1 ml ethanol. Leaf discs from Dp*CYP87A106*-RNAi *D. purpurea* plants (for example, lines 1 and 2) were used in feeding experiments. Briefly, 10 mm leaf discs (5–7 discs for each genotype) were cut using a borer, placed into 5 ml Eppendorf tubes with de-ionized water (~2 ml) and then fed with pregnenolone (final concentration, 0.32 mM). Leaf discs treated with de-ionized water were used as control. Eppendorf tubes with treated and control leaf discs were further incubated in a growth chamber at 25 °C, with 16:8 hour photoperiod under LED light. After 4–6 days, leaf discs were collected and analysed by LC–MS for the accumulated cardenolides or pathway intermediates as described above.

### Statistical analysis

Microsoft Excel 2016 and GraphPad Prism 8 software were used for regular statistical analysis. A two-tailed Student’s *t*-test was used to calculate significant differences among samples or genotypes. Details of biological or technical replicates and statistical parameters used in various experiments are provided in Methods and the captions of the figures and supplementary figures, wherever necessary.

### Reporting summary

Further information on research design is available in the Nature Portfolio Reporting Summary linked to this article.

### Supplementary information

Supplementary Information Supplementary Figs. 1–13.

Reporting Summary

Supplementary Data 1 CYP87A protein sequences from various plants used in construction of the phylogenetic analysis.

Supplementary Table 1 Oligonucleotides used in this study.

Supplementary Data Source data for supplementary figures.

### Source data

Source Data Figs. 2 and 3 Source data for Figs. 2c,d,g,h and 3c,d.

## Data Availability

Data supporting the findings of this work are available within the article and its Supplementary Information. *D. purpurea* and *C. procera* RNA-seq data associated with this article have been deposited into the NCBI Sequence Read Archive with BioProject identifiers PRJNA929980 and PRJNA1003839, respectively. The databases used in this study are UniProt Swiss-Prot (release 2022_02) and the Pfam database (Pfam-A.hmm release 34.0). Correspondence and requests for materials should be addressed to P.D.S. or S.E.O.’C. Source data are provided with this paper.
