# Supplementary material for: Promiscuous CYP87A enzyme activity initiates cardenolide biosynthesis in plants
Source: Nat Plants. 2023 Sep 18;9(10):1607–17. doi: 10.1038/s41477-023-01515-9 (PMC10581899; doi:10.1038/s41477-023-01515-9)
Supplement: Supplementary file 1 — Supplementary Figs. 1–13. [file 41477_2023_1515_MOESM1_ESM.pdf]

nature plants

Letter

https://doi.org/10.1038/s41477-023-01515-9

Promiscuous CYP87A enzyme activity initiates cardenolide biosynthesis in plants

In the format provided by the authors and unedited

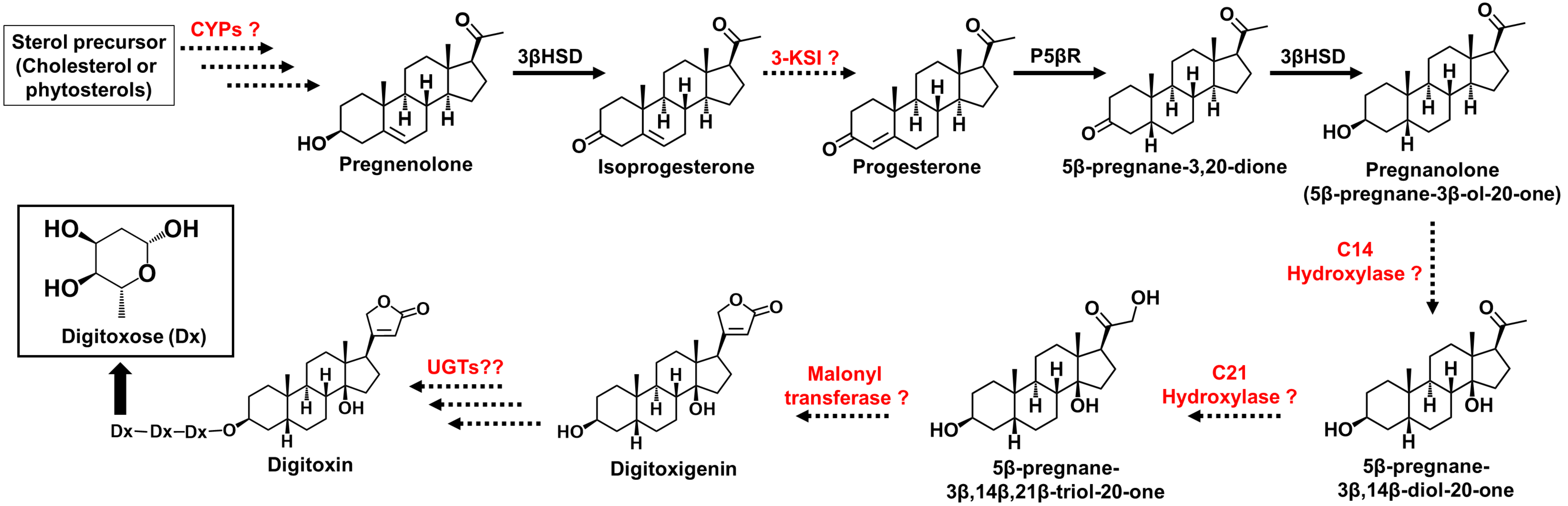

Sterol precursor (Cholesterol or phytosterols)
CYPs ?
Pregnenolone
3βHSD
Isoprogesterone
3-KSI ?
Progesterone
P5βR
5β-pregnane-3,20-dione
3βHSD
Pregnanolone (5β-pregnane-3β-ol-20-one)
C14 Hydroxylase ?
5β-pregnane-3β,14β-diol-20-one
C21 Hydroxylase ?
5β-pregnane-3β,14β,21β-triol-20-one
Malonyl transferase ?
Digitoxigenin
UGTs??
Digitoxin
Dx–Dx–Dx–O
Digitoxose (Dx)

Supplementary Fig. 1: **Cardenolide biosynthesis in *D. purpurea* plants.** Predicted steps towards formation of digitoxin from suggested sterol precursor are presented as a representative example. Formation of pregnenolone is the first committed step in cardenolide biosynthesis in plants. The unknown steps (dashed arrows) and corresponding hypothesized enzymes (in red) are shown. Known biosynthetic enzymes (solid arrows) are marked in black. 3βHSD, 3β-hydroxysteroid dehydrogenase; P5βR, progesterone 5β-reductase; 3-KSI, 3-ketosteroid isomerase; UGTs, UDP-glycosyltransferases.

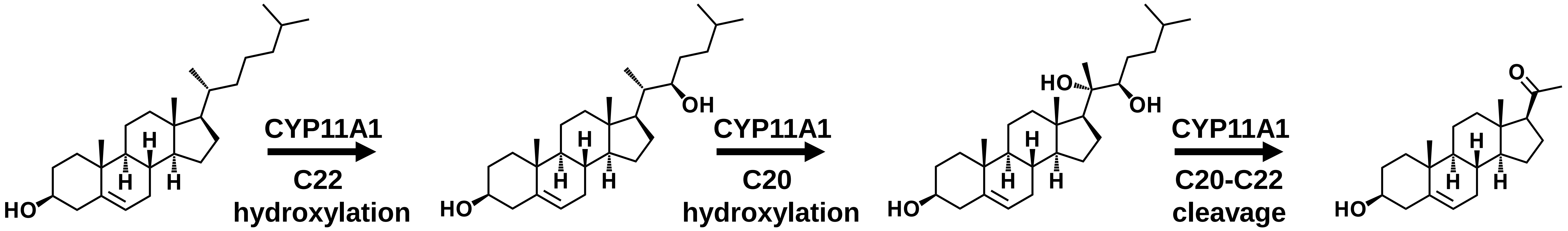

Cholesterol **22*R*-hydoxycholesterol** **20*R*,22*R*-dihydoxycholesterol** **Pregnenolone**

Supplementary Fig. 2: Three step reaction sequence catalyzed by CYP11A1 (solid black arrows) during conversion of cholesterol to pregnenolone.

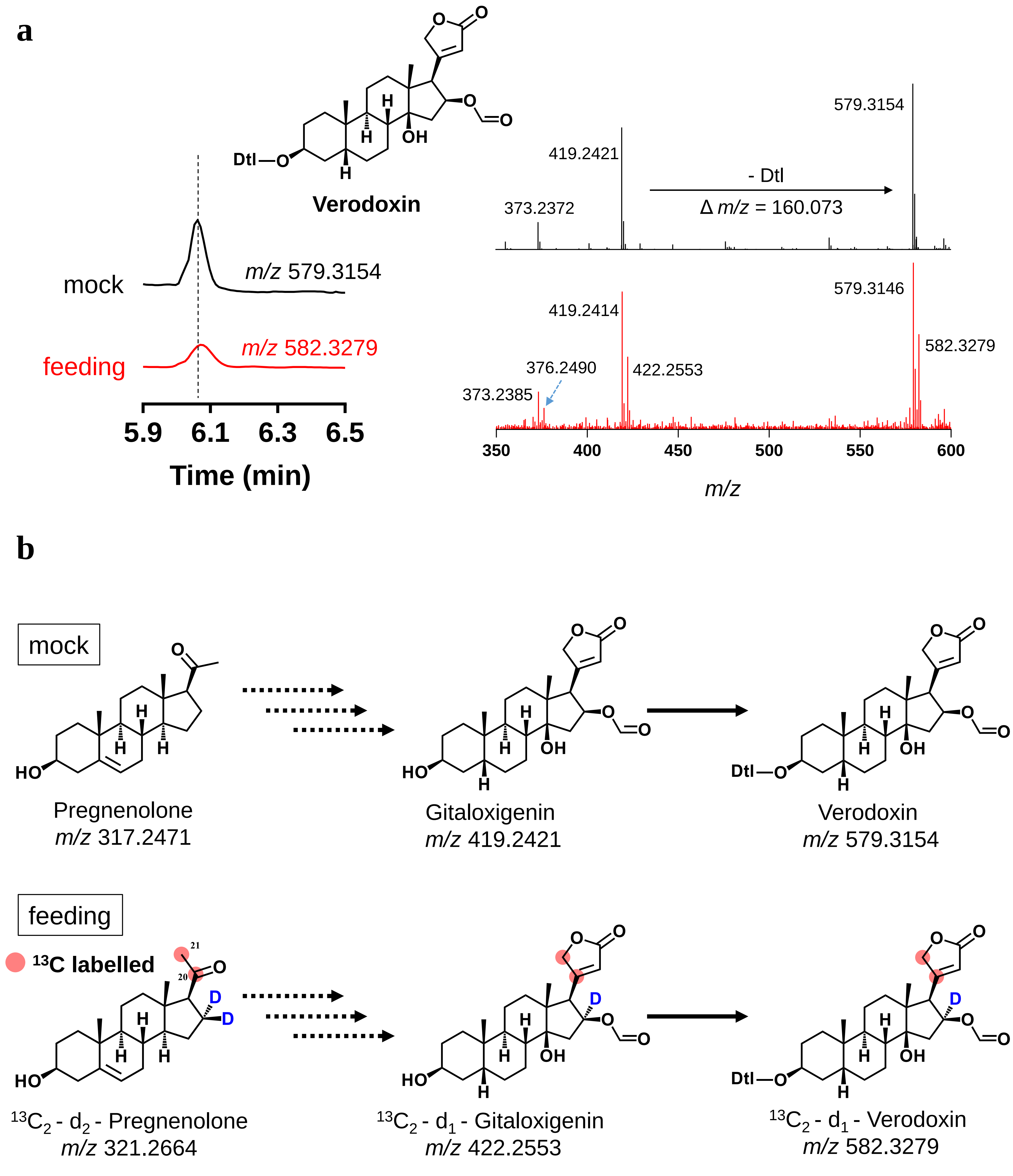
a
Verodoxin
mock
feeding
m/z 579.3154
m/z 582.3279
5.9
6.1
6.3
6.5
Time (min)
579.3154
419.2421
373.2372
- Dtl
Δ m/z = 160.073
579.3146
419.2414
582.3279
376.2490
422.2553
373.2385
350
400
450
500
550
600
m/z
b
mock
Pregnenolone
m/z 317.2471
Gitaloxigenin
m/z 419.2421
Verodoxin
m/z 579.3154
feeding
13C labelled
13C2 - d2 - Pregnenolone
m/z 321.2664
13C2 - d1 - Gitaloxigenin
m/z 422.2553
13C2 - d1 - Verodoxin
m/z 582.3279

Supplementary Fig. 3: **Incorporation of pregnenolone to verodoxin as demonstrated by stable isotope labelled feeding studies.** LC-MS analysis of verodoxin after feeding of labelled pregnenolone ($^{13}C_2$(20,21)-16,16-D_2-pregnenolone) to in vitro grown 4-week old *D. purpurea*. **(a)** Comparison of extracted ion chromatograms (left) and corresponding MS/MS fragmentation spectra (right) in feeding (treated with labelled pregnenolone, in red) and mock (treated with deionized water, in black) samples respectively. **(b)** Structures of putative ion fragments generated in MS/MS analysis for verdoxin in mock and feeding samples. Mass to charge (*m/z*) ions are indicated for verodoxin under feeding and mock treatments. Dtl: digitalose.

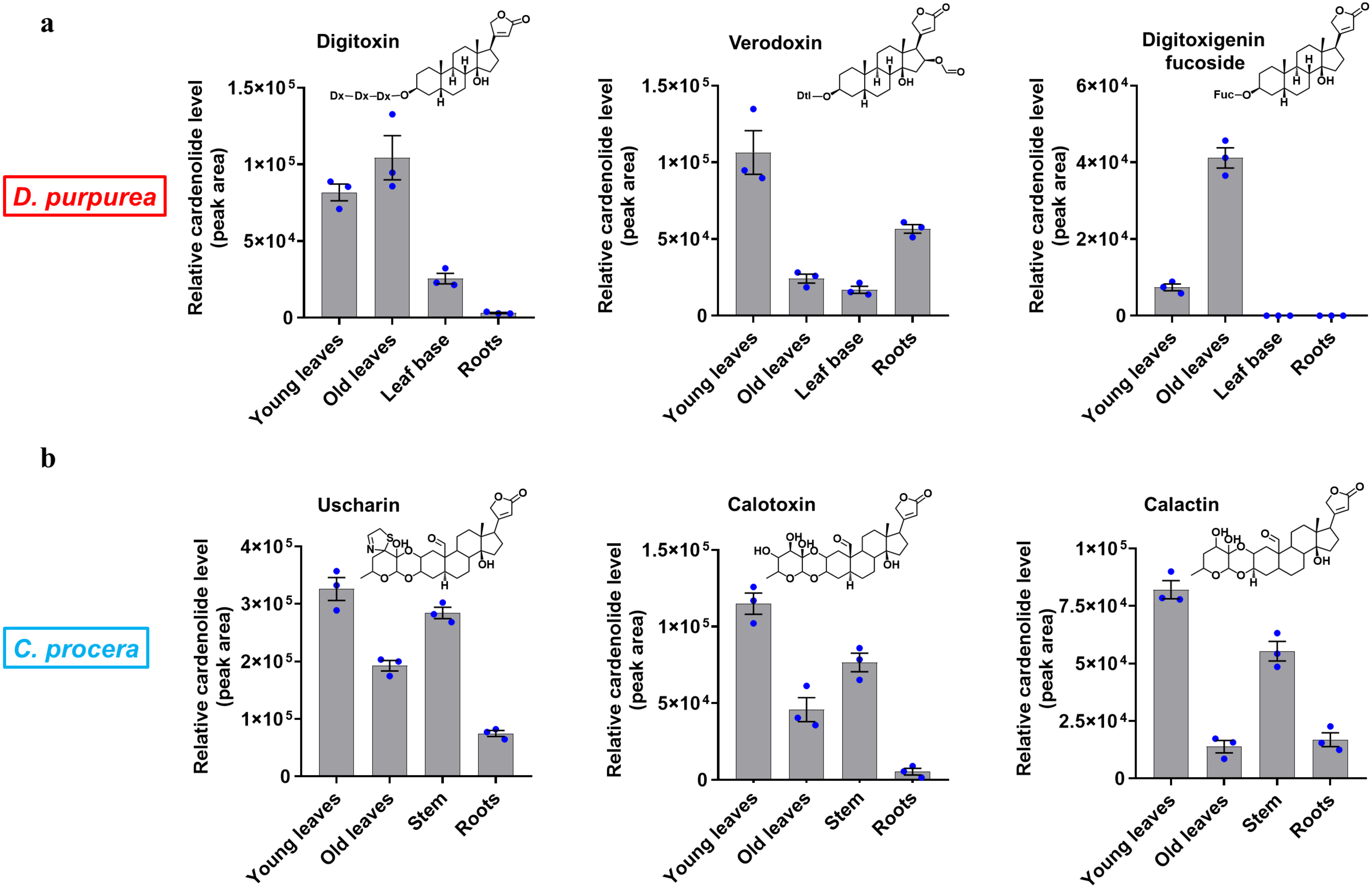

a
D. purpurea
Digitoxin
Verodoxin
Digitoxigenin fucoside
Relative cardenolide level (peak area)
Young leaves
Old leaves
Leaf base
Roots
b
C. procera
Uscharin
Calotoxin
Calactin
Stem

Supplementary Fig. 4: **LC-MS based profiling of selected cardenolides in different tissues of *D. purpurea* (a) and *C. procera* (b).** The peak areas for each cardenolide were determined using the Bruker Compass Data Analysis (Version 5.3) software. The values indicate means of biological replicates ± standard error mean (n=3). Dx: digitoxose; Dtl: digitalose; Fuc: fucose.

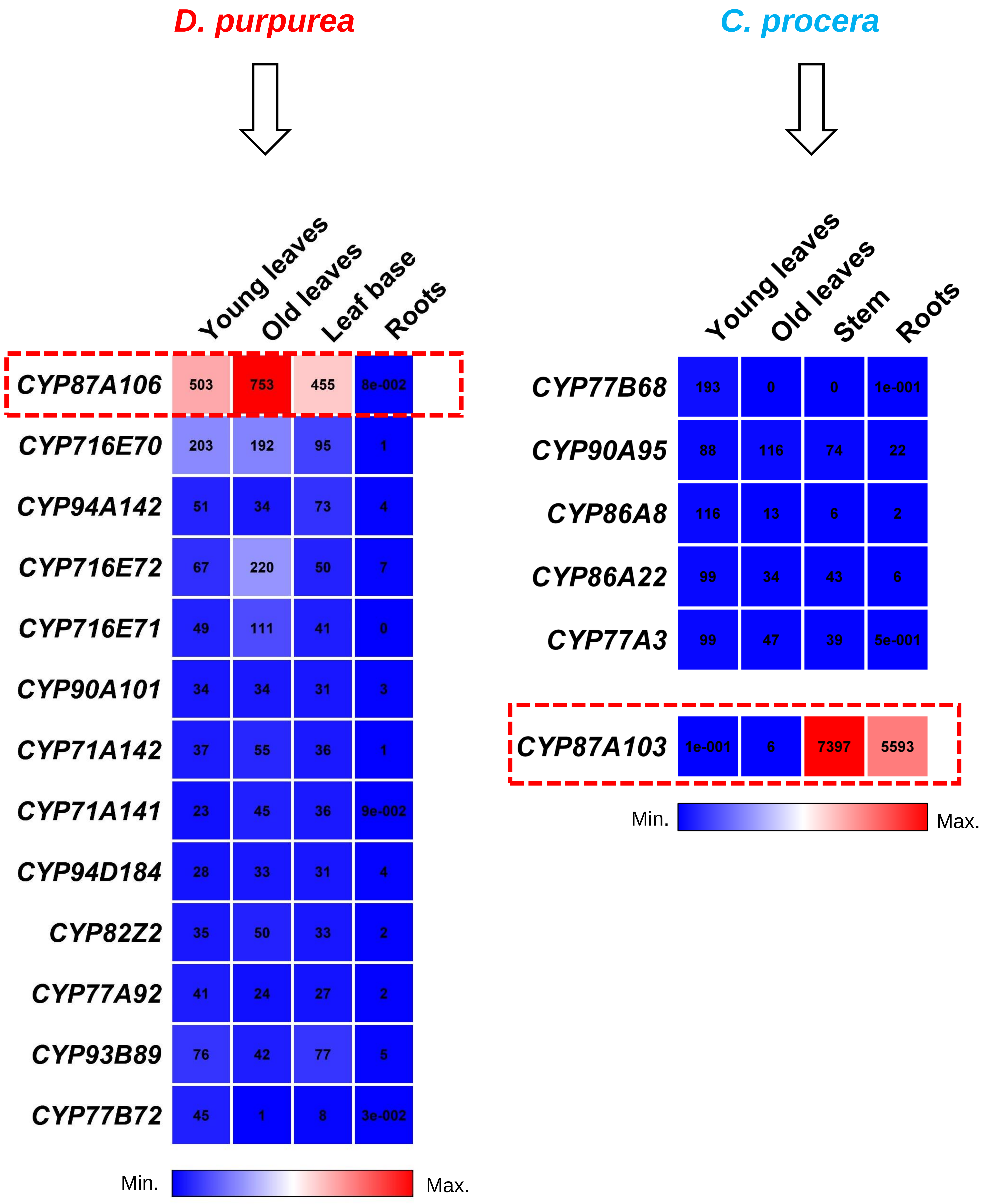

D. purpurea
C. procera
Young leaves
Old leaves
Leaf base
Roots
CYP87A106 503 753 455 8e-002
CYP716E70 203 192 95 1
CYP94A142 51 34 73 4
CYP716E72 67 220 50 7
CYP716E71 49 111 41 0
CYP90A101 34 34 31 3
CYP71A142 37 55 36 1
CYP71A141 23 45 36 9e-002
CYP94D184 28 33 31 4
CYP82Z2 35 50 33 2
CYP77A92 41 24 27 2
CYP93B89 76 42 77 5
CYP77B72 45 1 8 3e-002
Min.
Max.
Young leaves
Old leaves
Stem
Roots
CYP77B68 193 0 0 1e-001
CYP90A95 88 116 74 22
CYP86A8 116 13 6 2
CYP86A22 99 34 43 6
CYP77A3 99 47 39 5e-001
CYP87A103 1e-001 6 7397 5593
Min.
Max.

Supplementary Fig. 5: **Expression profile of candidate CYP genes in *D. purpurea* (left) and *C. procera* (right) different tissue types (RNA-seq expression data).** Normalized TPM (transcripts per million mapped reads) values were used to infer the expression profile. Two CYP candidates, CYP87A106 (*D. purpurea*) and CYP87A103 (*C. procera*) characterized further in this study are marked in dashed red rectangle.

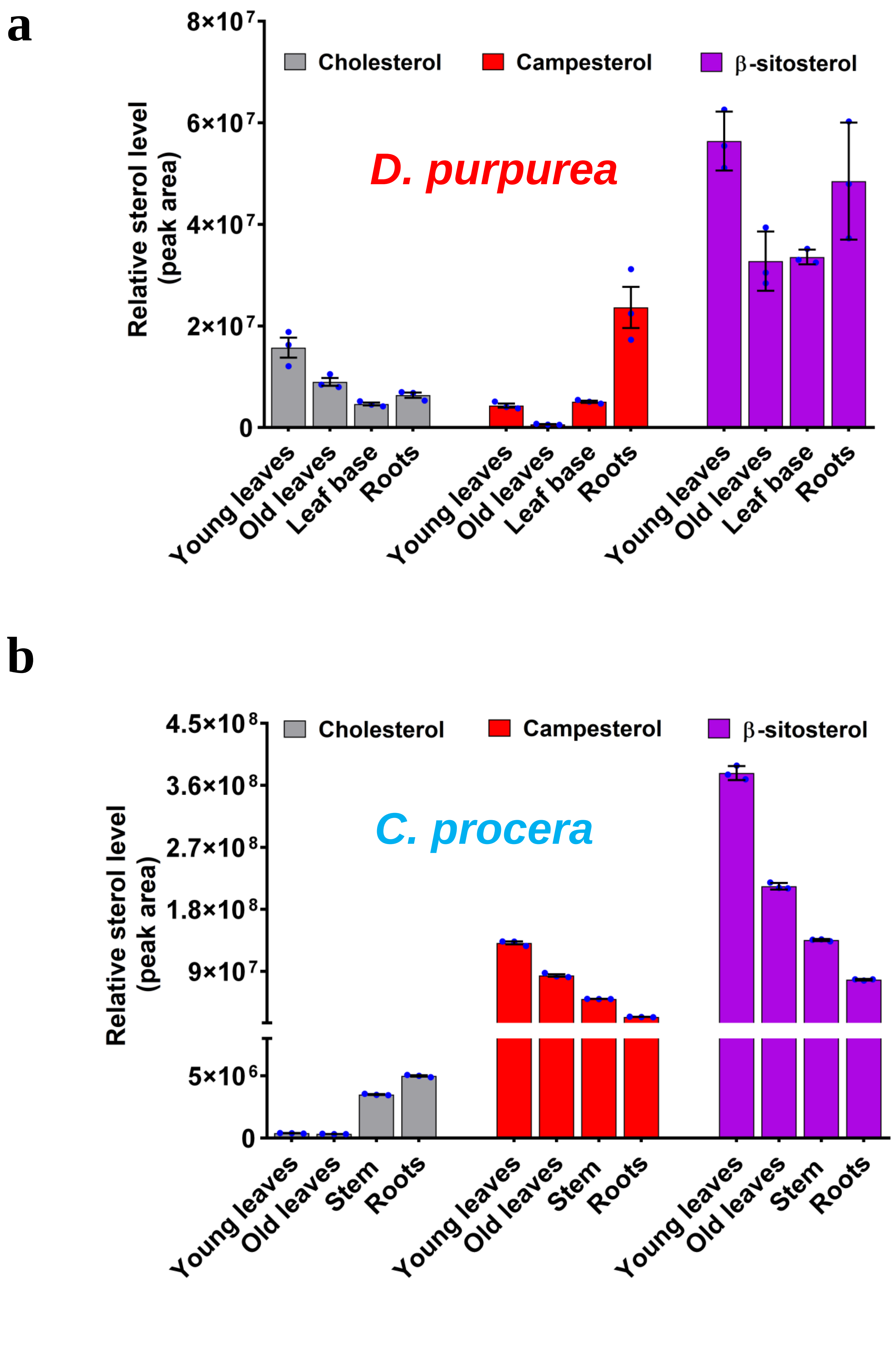
a
8×10^7
6×10^7
4×10^7
2×10^7
0
Relative sterol level (peak area)
Cholesterol
Campesterol
β-sitosterol
D. purpurea
Young leaves
Old leaves
Leaf base
Roots
b
4.5×10^8
3.6×10^8
2.7×10^8
1.8×10^8
9×10^7
5×10^6
0
C. procera
Stem

Supplementary Fig. 6: **Levels of cholesterol and phytosterols in different tissues of *D. purpurea* (a) and *C. procera* (b) as determined by GC-MS.** The values indicate means of biological replicates ± standard error mean (n=3) obtained from three different plants.

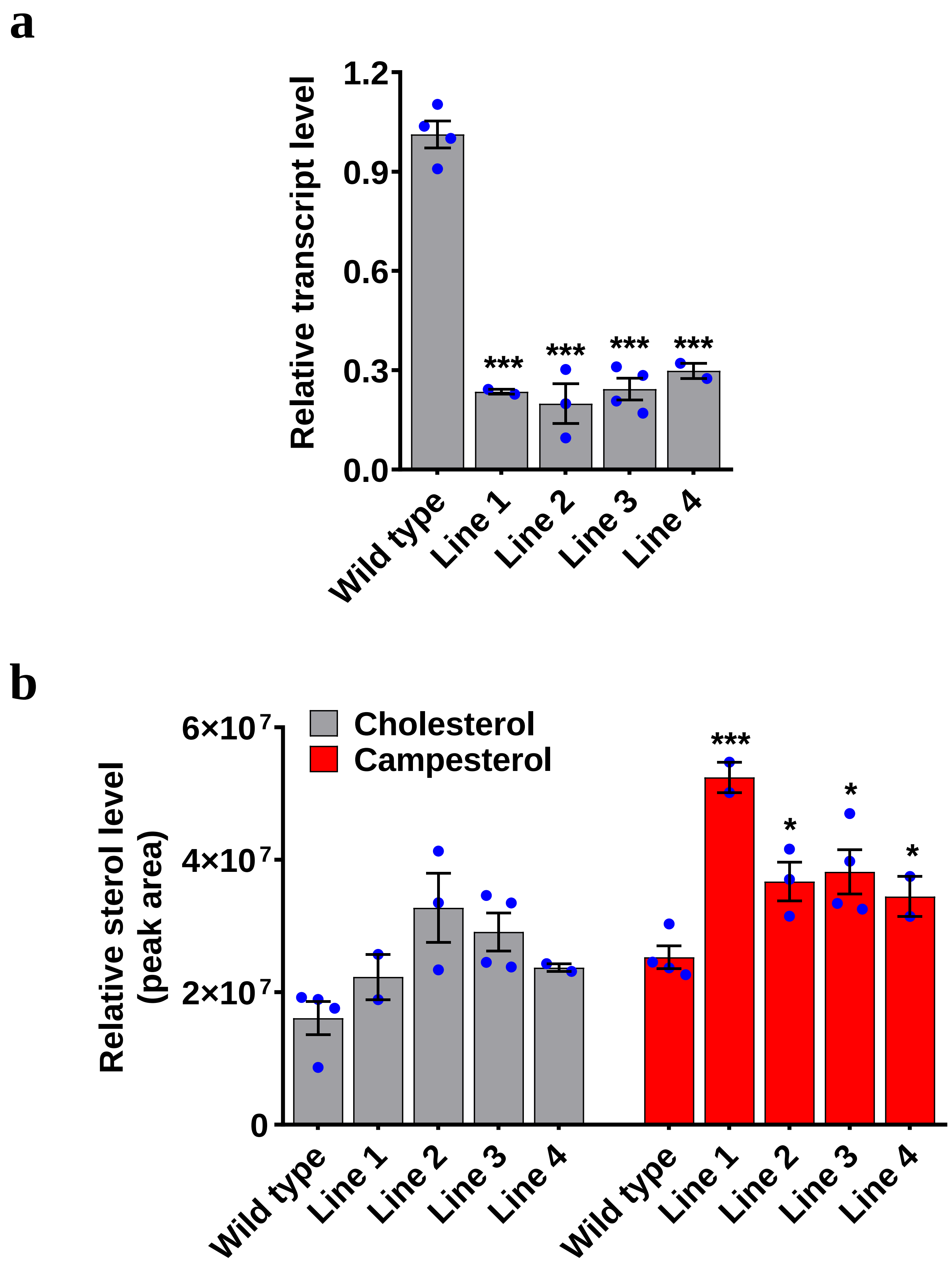
a
Relative transcript level
1.2
0.9
0.6
0.3
0.0
***
***
***
***
Wild type
Line 1
Line 2
Line 3
Line 4
b
Cholesterol
Campesterol
Relative sterol level
(peak area)
6×10^7
4×10^7
2×10^7
0
***
*
*
*
Wild type
Line 1
Line 2
Line 3
Line 4
Wild type
Line 1
Line 2
Line 3
Line 4

Supplementary Fig. 7: **Dp*CYP87A106* expression (a) and levels of cholesterol and campesterol (b) in leaves of Dp*CYP87A106*-silenced *D. purpurea* lines (by RNAi) compared to wild type ones.** Four independent Dp*CYP87A106*-RNAi transgenic *D. purpurea* lines (1, 2, 3 and 4) were generated after stable transformation. Values indicate means ± standard error mean (n=4 for wild type and n ≥ 2 for individual transgenic lines genotype). Asterisks indicate significant changes compared to wild type samples as calculated by a two-tailed Student's t-test (*P-value < 0.05; **P-value < 0.01; ***P-value < 0.001). GC-MS was used for sterol profiling.

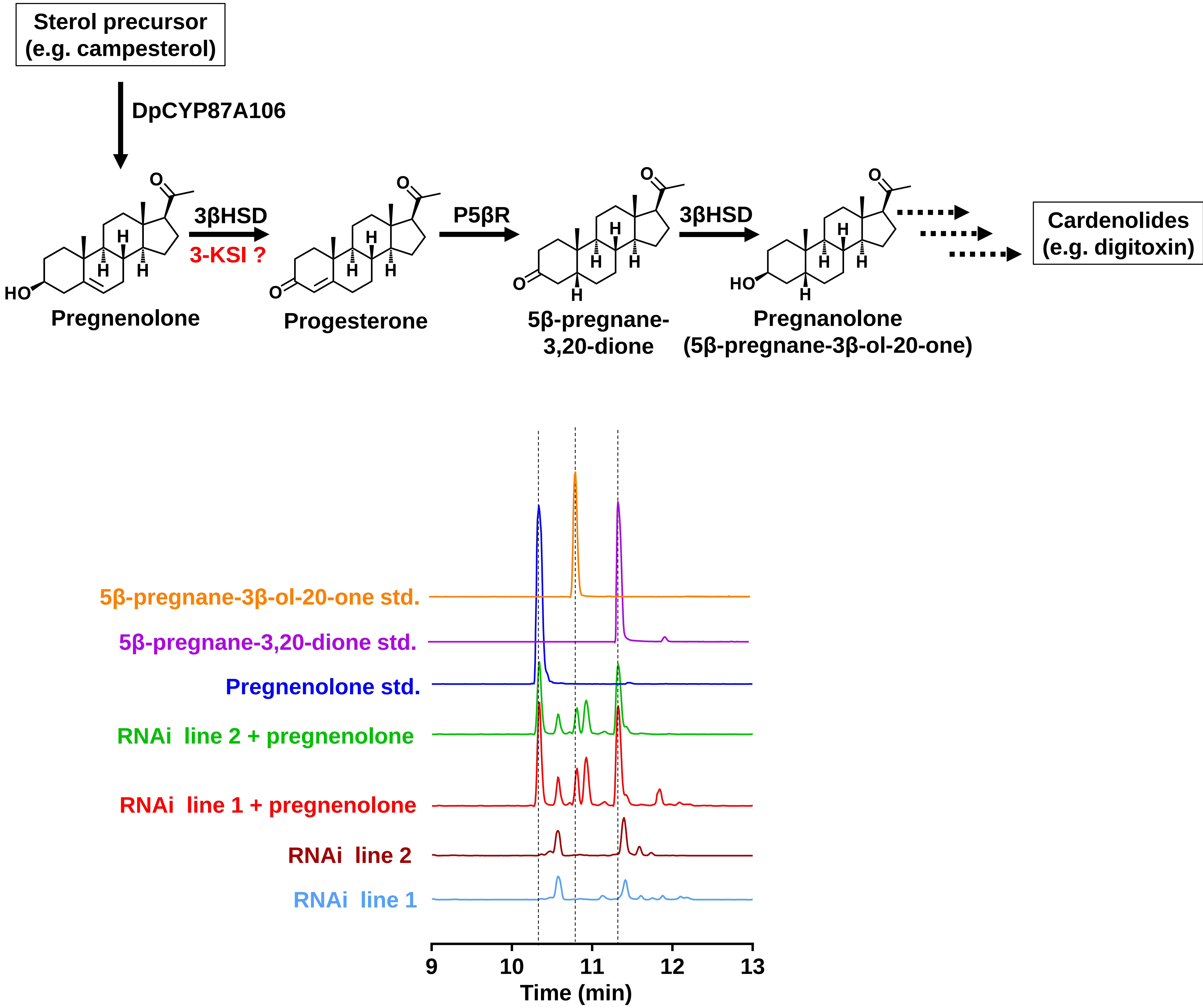

Sterol precursor (e.g. campesterol)
DpCYP87A106
Pregnenolone
3βHSD
3-KSI ?
Progesterone
P5βR
5β-pregnane-3,20-dione
3βHSD
Pregnanolone (5β-pregnane-3β-ol-20-one)
Cardenolides (e.g. digitoxin)
5β-pregnane-3β-ol-20-one std.
5β-pregnane-3,20-dione std.
Pregnenolone std.
RNAi line 2 + pregnenolone
RNAi line 1 + pregnenolone
RNAi line 2
RNAi line 1
9
10
11
12
13
Time (min)

Supplementary Fig. 8: **Accumulation of cardenolide pathway intermediates (downstream of pregnenolone) following feeding of pregnenolone to Dp*CYP87A106*-silenced *D. purpurea* lines.** Aligned extracted ion chromatograms (LC-MS) are shown for feeding (RNAi line 1 and 2 treated with pregnenolone) and control (RNAi line 1 and 2 treated with water) along with authentic sterol standards (pregnenolone, 5β-pregnane-3,20-dione and 5β-pregnane-3β-ol-20-one). Cut leaf disks obtained from each genotype were used in the feeding experiments. Simplified cardenolide pathway from sterol precursor is shown in the upper panel. Formation of pregnenolone catalyzed by the DpCYP87A106 is the first committed step in cardenolide biosynthesis. Known biosynthetic enzymes (in black) and hypothesized ones (in red) are marked in pathway scheme. 3βHSD, 3β-hydroxysteroid dehydrogenase; P5βR, progesterone 5β-reductase; 3-KSI, 3-ketosteroid isomerase.

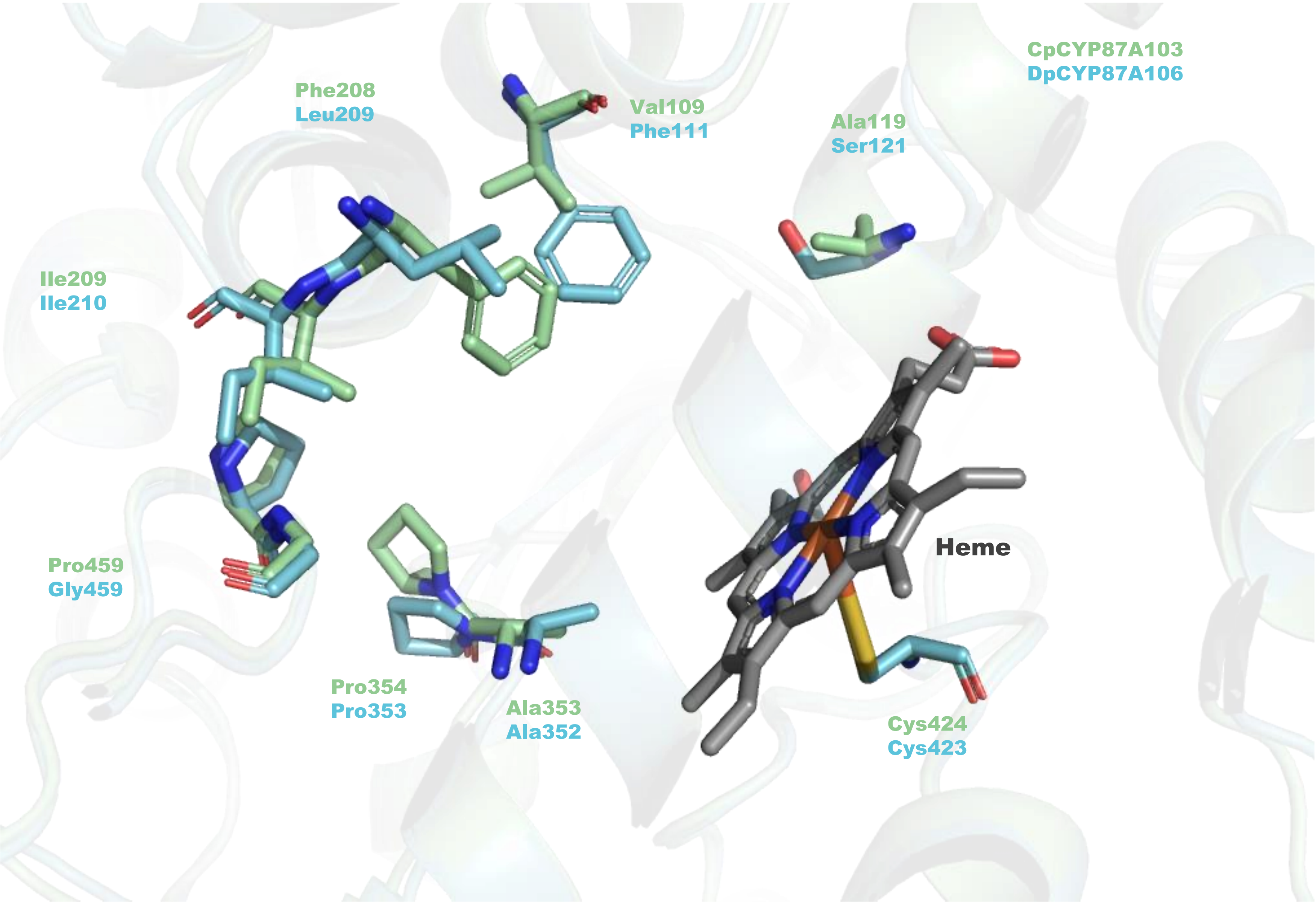

CpCYP87A103
DpCYP87A106
Phe208
Leu209
Val109
Phe111
Ala119
Ser121
Ile209
Ile210
Pro459
Gly459
Heme
Pro354
Pro353
Ala353
Ala352
Cys424
Cys423

Supplementary Fig. 9: Comparison of active site of CpCYP87A103 (green) and DpCYP87A106 (blue) docked with heme-thiolate cofactor.

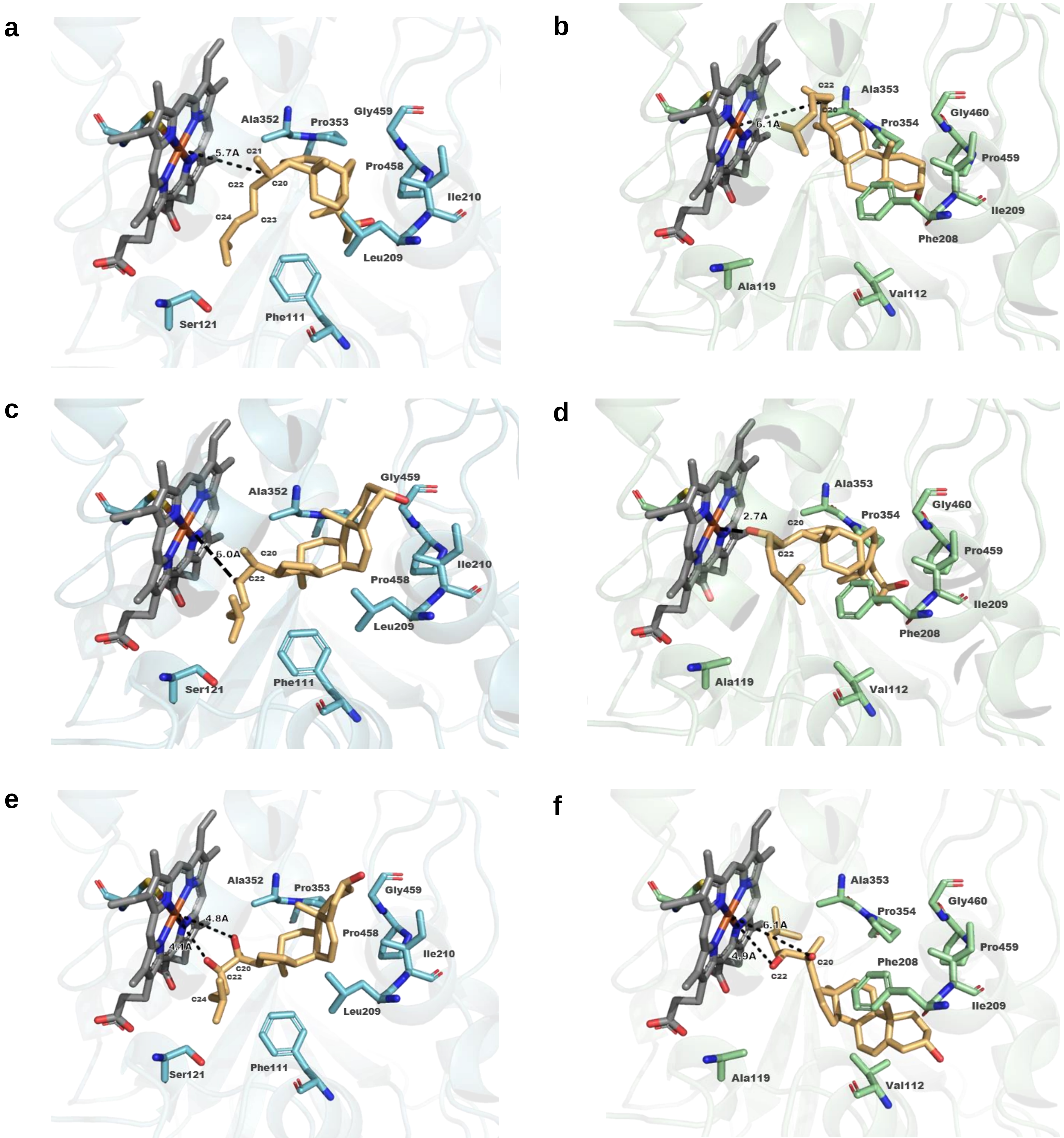
a
Ala352
Pro353
Gly459
5.7A
C21
C22
C20
Pro458
Ile210
C24
C23
Leu209
Ser121
Phe111
b
C22
Ala353
C20
6.1A
Pro354
Gly460
Pro459
Ile209
Phe208
Ala119
Val112
c
Gly459
Ala352
6.0A
C20
C22
Pro458
Ile210
Leu209
Ser121
Phe111
d
Ala353
Pro354
Gly460
2.7A
C20
C22
Pro459
Ile209
Phe208
Ala119
Val112
e
Ala352
Pro353
Gly459
4.8A
4.1A
Pro458
C20
C22
Ile210
C24
Leu209
Ser121
Phe111
f
Ala353
Gly460
Pro354
6.1A
4.9A
C20
C22
Pro459
Phe208
Ile209
Ala119
Val112

Supplementary Fig. 10: Docking of cholesterol (a and b), 22-hydroxycholesterol (c and d) and 20,22-dihydroxycholesterol (e and f) into the active site of DpCYP87A106 and CpCYP87A103 respectively. DpCYP87A106 and CpCYP87A103 homology models docked with heme-thiolate cofactor. Dashes represent distance between carbon undergoing hydroxylation and heme in Angstrom.

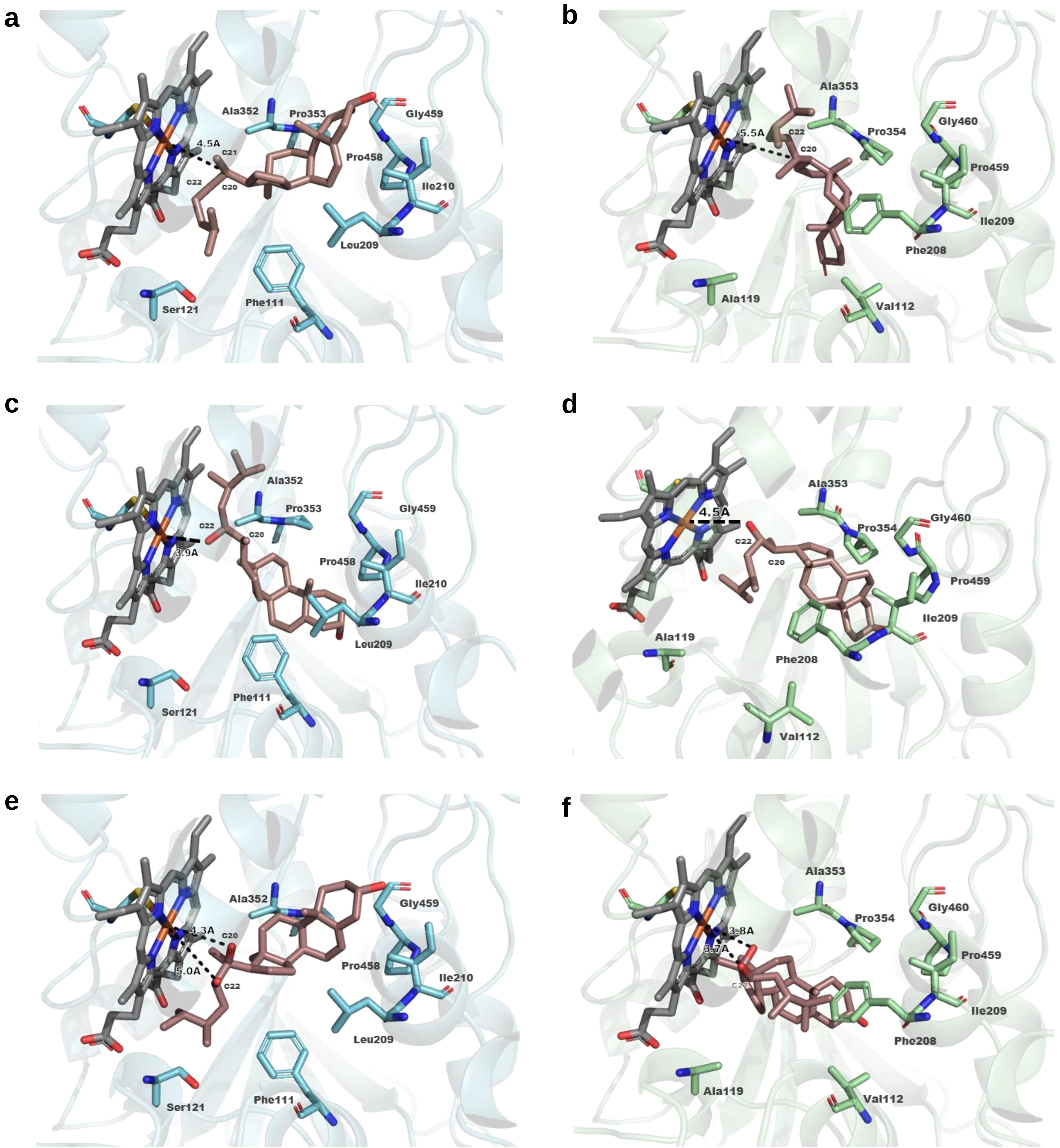
a
4.5A
c21
c22
c20
Ala352
Pro353
Gly459
Pro458
Ile210
Leu209
Ser121
Phe111
b
5.5A
c22
c20
Ala353
Pro354
Gly460
Pro459
Ile209
Phe208
Ala119
Val112
c
c22
c20
3.9A
Ala352
Pro353
Gly459
Pro458
Ile210
Leu209
Ser121
Phe111
d
4.5A
c22
c20
Ala353
Pro354
Gly460
Pro459
Ile209
Phe208
Ala119
Val112
e
4.3A
c20
5.0A
c22
Ala352
Gly459
Pro458
Ile210
Leu209
Ser121
Phe111
f
3.8A
3.7A
Ala353
Pro354
Gly460
Pro459
Ile209
Phe208
Ala119
Val112

Supplementary Fig. 11: Docking of campesterol (a and b), 22-hydroxycampesterol (c and d) and 20,22-dihydroxycampesterol (e and f) into the active site of DpCYP87A106 and CpCYP87A103 respectively. DpCYP87A106 and CpCYP87A103 homology models docked with heme-thiolate cofactor. Dashes represent distance between carbon undergoing hydroxylation and heme in Angstrom.

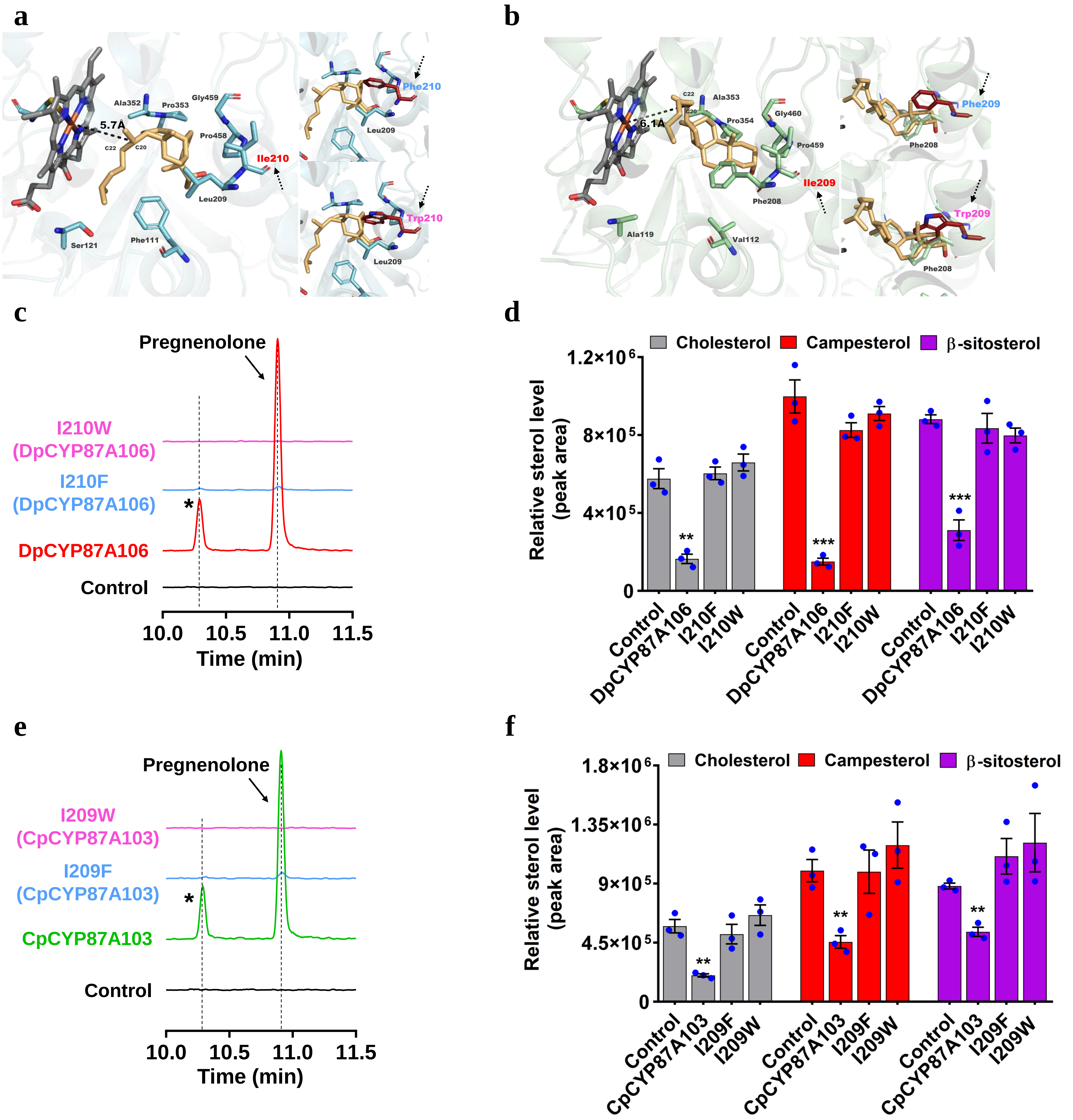

a
5.7Å
C22
C20
Ala352
Pro353
Gly459
Pro458
Ile210
Leu209
Ser121
Phe111
Phe210
Leu209
Trp210
Leu209
b
C22
C20
6.1Å
Ala353
Pro354
Gly460
Pro459
Ile209
Phe208
Ala119
Val112
Phe209
Phe208
Trp209
Phe208
c
Pregnenolone
I210W (DpCYP87A106)
I210F (DpCYP87A106)
*
DpCYP87A106
Control
10.0 10.5 11.0 11.5
Time (min)
d
Cholesterol
Campesterol
β-sitosterol
Relative sterol level (peak area)
1.2×10^6
8×10^5
4×10^5
0
**
***
***
Control DpCYP87A106 I210F I210W
Control DpCYP87A106 I210F I210W
Control DpCYP87A106 I210F I210W
e
Pregnenolone
I209W (CpCYP87A103)
I209F (CpCYP87A103)
*
CpCYP87A103
Control
10.0 10.5 11.0 11.5
Time (min)
f
Cholesterol
Campesterol
β-sitosterol
Relative sterol level (peak area)
1.8×10^6
1.35×10^6
9×10^5
4.5×10^5
0
**
**
**
Control CpCYP87A103 I209F I209W
Control CpCYP87A103 I209F I209W
Control CpCYP87A103 I209F I209W

Supplementary Fig. 12: Role of Ile210/Ile209 residue in DpCYP87A106 and CpCYP87A103 catalyzed pregnenolone formation. (a, b) DpCYP87A106 (a) and CpCYP87A103 (b) docked with thiolate-heme and cholesterol (on left side of each panel). Dashed line shows distance of C20 to heme-thiolate cofactor. Active site of DpCYP87A106 (a) and CpCYP87A103 (b) showing steric hindrance of cholesterol substrate by Ile210Phe (upper, on right side of each panel) and Ile210Trp (lower, on right side of each panel) mutants. **(c, d)** pregnenolone (c) and major sterols (d) profiling in I210F and I210W mutants of DpCYP87A106 following transient expression in *N. benthamiana* leaves. **(e, f)** pregnenolone (e) and major sterols (f) profiling in I209F and I209W mutants of CpCYP87A103 following transient expression in *N. benthamiana* leaves. Leaves infiltrated with empty vector were used as a negative control (panel c and e, in black), while leaves infiltrated with wild type DpCYP87A106 (panel c, in red) or CpCYP87A103 (panel e, in green) were used as positive control. Asterisk (*) shows the presence of a new compound at different retention time, having same mass spectrum as that of pregnenolone (see Fig. 2b). The values in panel (d) and (f) indicate the mean of three biological replicates ± standard error mean (n=3). Asterisks indicate significant changes compared to control samples as calculated by two tailed Student's t-test (*P-value < 0.05; **P-value < 0.01; ***P-value < 0.001). Sterol profiling was done by GC-MS.

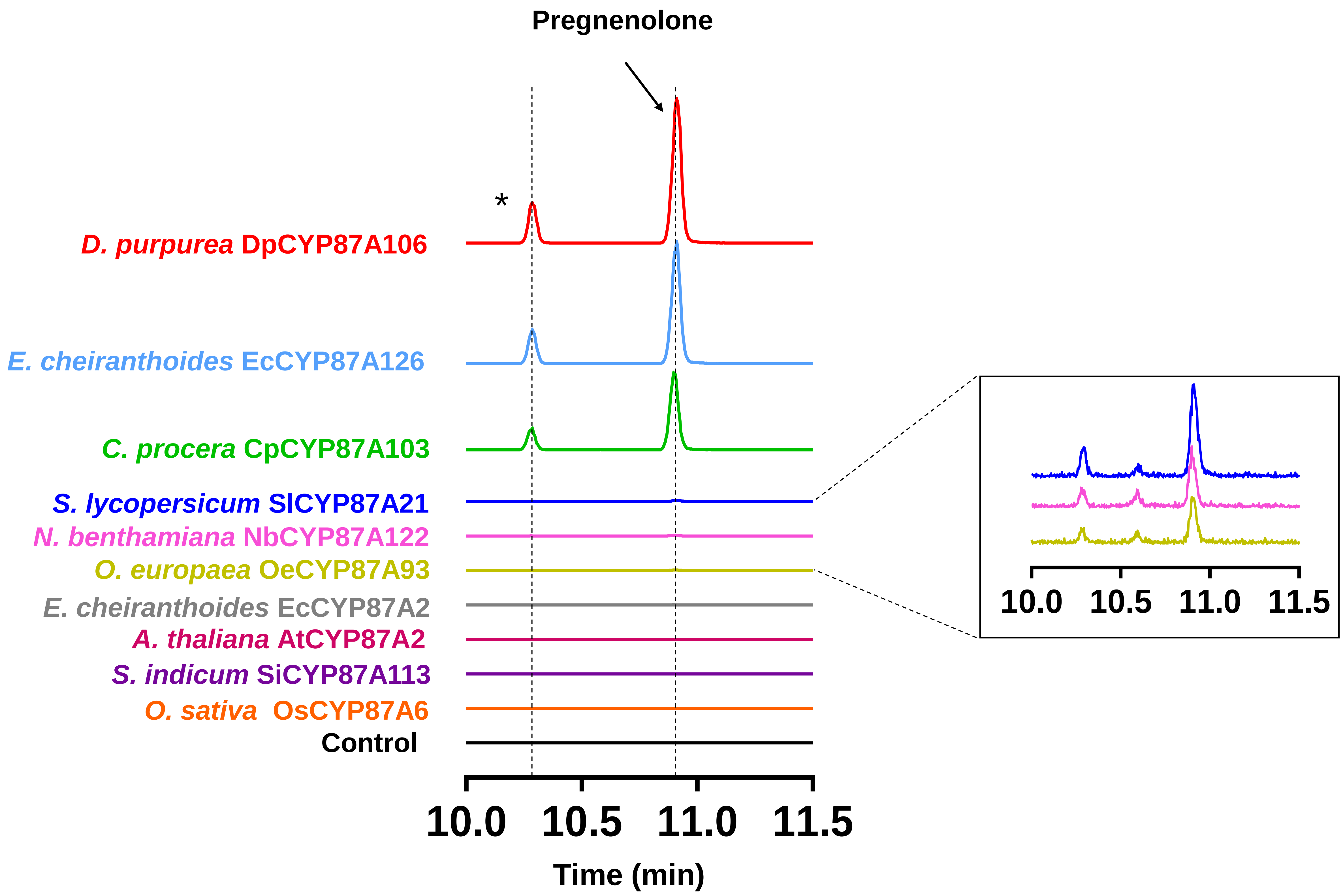

Pregnenolone
*
D. purpurea DpCYP87A106
E. cheiranthoides EcCYP87A126
C. procera CpCYP87A103
S. lycopersicum SlCYP87A21
N. benthamiana NbCYP87A122
O. europaea OeCYP87A93
E. cheiranthoides EcCYP87A2
A. thaliana AtCYP87A2
S. indicum SiCYP87A113
O. sativa OsCYP87A6
Control
10.0
10.5
11.0
11.5
Time (min)
10.0
10.5
11.0
11.5

Supplementary Fig. 13: Aligned extracted ion chromatograms (GC-MS) of pregnenolone (*m/z* 388.2, trimethylsilylated) from *N. benthamiana* leaves extracts transiently expressing *CYP87A* genes from either cardenolide producing or cardenolide free plants. Transient expression of three *CYP87A* genes from three cardenolide producers, namely EcCYP87A126 (*E. cheiranthoides*), DpCYP87A106 (*D. purpurea*) and CpCYP87A103 (*C. procera*) in *N. benthamiana* leaves produce high levels of pregnenolone, that typically does not accumulate in *N. benthamiana*. Leaves infiltrated with empty vector were used as a control (shown in black). Sterol profiling was done by GC-MS. Asterisk (*) shows the presence of a new compound at different retention time, having same mass spectrum as that of pregnenolone (see Fig. 2b).
