# Supplementary material for: Promiscuous CYP87A enzyme activity initiates cardenolide biosynthesis in plants
Source: Nat Plants. 2023 Sep 18;9(10):1607–17. doi: 10.1038/s41477-023-01515-9 (PMC10581899; doi:10.1038/s41477-023-01515-9)
Supplement: Supplementary file 2 — Reporting Summary [file 41477_2023_1515_MOESM2_ESM.pdf]

nature portfolio

Corresponding author(s): Dr. Prashant Sonawane
Prof. Sarah O'Connor

Last updated by author(s): 11 August 2023

Reporting Summary

Nature Portfolio wishes to improve the reproducibility of the work that we publish. This form provides structure for consistency and transparency in reporting. For further information on Nature Portfolio policies, see our Editorial Policies and the Editorial Policy Checklist.

Statistics

For all statistical analyses, confirm that the following items are present in the figure legend, table legend, main text, or Methods section.

n/a	Confirmed	
☐	☒	The exact sample size (*n*) for each experimental group/condition, given as a discrete number and unit of measurement
☐	☒	A statement on whether measurements were taken from distinct samples or whether the same sample was measured repeatedly
☐	☒	The statistical test(s) used AND whether they are one- or two-sided<br>*Only common tests should be described solely by name; describe more complex techniques in the Methods section.*
☒	☐	A description of all covariates tested
☒	☐	A description of any assumptions or corrections, such as tests of normality and adjustment for multiple comparisons
☐	☒	A full description of the statistical parameters including central tendency (e.g. means) or other basic estimates (e.g. regression coefficient) AND variation (e.g. standard deviation) or associated estimates of uncertainty (e.g. confidence intervals)
☐	☒	For null hypothesis testing, the test statistic (e.g. *F*, *t*, *r*) with confidence intervals, effect sizes, degrees of freedom and *P* value noted<br>*Give P values as exact values whenever suitable.*
☒	☐	For Bayesian analysis, information on the choice of priors and Markov chain Monte Carlo settings
☒	☐	For hierarchical and complex designs, identification of the appropriate level for tests and full reporting of outcomes
☒	☐	Estimates of effect sizes (e.g. Cohen's *d*, Pearson's *r*), indicating how they were calculated

Our web collection on statistics for biologists contains articles on many of the points above.

Software and code

Policy information about availability of computer code

Data collection	All presented data have been acquired using existing and routinely used softwares. These were mentioned in the respective parts of manuscript. LC-MS data presented in this study was collected and analyzed using Thermo Scientific UltiMate 3000 ultra-high performance liquid chromatography (UHPLC) system coupled to an Impact II UHR-Q-ToF (Ultra-High Resolution Quadrupole-Time-of-Flight) mass spectrometer (Bruker Daltonics). GC-MS data was collected and analyzed using the GC–MS system comprised a GC PAL autosampler (CTC Analytics), a trace 1310 GC ultra-gas chromatograph equipped with a split-splitless injector and ISQ quadrupole MS (Thermo Scientific).
Data analysis	Bruker Compass Data Analysis (Version 5.3) and Xcalibur (Thermo Scientific version 4.2.47) softwares were used for LC-MS and GC-MS data analysis respectively. Microsoft Excel 2016 and GraphPad Prism 8 softwares were used for statistical analysis. Chemical structures were generated in ChemDraw Professional 17.1. Phylogenetic tree was constructed using MEGAX (version 10.2.2). Primer Blast (NCBI) was used for designing qRT-PCR primers. AlphaFold2 and Webina were used for protein modeling and docking studies.

For manuscripts utilizing custom algorithms or software that are central to the research but not yet described in published literature, software must be made available to editors and reviewers. We strongly encourage code deposition in a community repository (e.g. GitHub). See the Nature Portfolio guidelines for submitting code & software for further information.

Data

Policy information about availability of data

All manuscripts must include a data availability statement. This statement should provide the following information, where applicable:

- Accession codes, unique identifiers, or web links for publicly available datasets
- A description of any restrictions on data availability
- For clinical datasets or third party data, please ensure that the statement adheres to our policy

Data supporting the findings of this work are available within the paper and its Supplementary Information files. Source data are provided with this paper. D. purpurea and C. procera RNA-seq data associated with this manuscript have been deposited into the NCBI Sequence Read Archive with BioProject IDs PRJNA929980 and PRJNA1003839, respectively. The databases used in this study are Uniprot Swiss-Prot (release 2022_02) and the Pfam database (Pfam-A.hmm release 34.0). Correspondence and requests for materials should be addressed to P.D.S. or S.E.O'C.

Human research participants

Policy information about studies involving human research participants and Sex and Gender in Research.

Reporting on sex and gender	Not applicable to our study
Population characteristics	Not applicable to our study
Recruitment	Not applicable to our study
Ethics oversight	Not applicable to our study

Note that full information on the approval of the study protocol must also be provided in the manuscript.

Field-specific reporting

Please select the one below that is the best fit for your research. If you are not sure, read the appropriate sections before making your selection.

☒ Life sciences ☐ Behavioural & social sciences ☐ Ecological, evolutionary & environmental sciences

For a reference copy of the document with all sections, see nature.com/documents/nr-reporting-summary-flat.pdf

Life sciences study design

All studies must disclose on these points even when the disclosure is negative.

Sample size	Statistical based sample size determination was not applicable to our study. Details of biological/technical replicates related to various experiments are provided in the paper, wherever necessary. Minimum of three biological replicates (except in some cases of transgenic analysis performed in T0 generation) were used while performing the experiments. Sample size information for each experiment is present in the paper.
Data exclusions	No data was excluded in our analysis
Replication	Details of biological/technical replicates used in various experiments are provided in methods section as well as in Main Figures and Supplementary Figures legends, wherever necessary.
Randomization	Minimum three independently grown N. benthamiana, D. purpurea and C. procera plants were chosen randomly for experiments and analysis. For yeast expression studies, positive transformants were selected based on colony PCR experiments for each characterized gene.
Blinding	Not applicable. Same treatment/parameter was used while comparing control/wild type versus treated/transgenic samples in experiments

Reporting for specific materials, systems and methods

We require information from authors about some types of materials, experimental systems and methods used in many studies. Here, indicate whether each material, system or method listed is relevant to your study. If you are not sure if a list item applies to your research, read the appropriate section before selecting a response.

Materials & experimental systems

n/a	Involved in the study
☒	☐ Antibodies
☒	☐ Eukaryotic cell lines
☒	☐ Palaeontology and archaeology
☒	☐ Animals and other organisms
☒	☐ Clinical data
☒	☐ Dual use research of concern

Methods

n/a	Involved in the study
☒	☐ ChIP-seq
☒	☐ Flow cytometry
☒	☐ MRI-based neuroimaging
