# Supplementary material for: Promiscuous CYP87A enzyme activity initiates cardenolide biosynthesis in plants
Source: Nat Plants. 2023 Sep 18;9(10):1607–17. doi: 10.1038/s41477-023-01515-9 (PMC10581899; doi:10.1038/s41477-023-01515-9)
Supplement: Supplementary file 3 — CYP87A protein sequences from various plants used in construction of the phylogenetic analysis. [file 41477_2023_1515_MOESM3_ESM.pdf]

Supplementary Data 1: CYP87A protein sequences from various plants used in construction of phylogenetic analysis.

> *Solanum lycopersicum* (SlCYP87A21)
MISVGMSIGAFLILIIIHWVYNWRNPRCNGKLPPGSMGWPLLGETIQFFTPNTTLDIAPF
VKERMKRYGPIFRTSVVGRPVIVSTDPDLNYFIFQQEGQSFQSWYPDTFTEIFGRQNV
GSLHGFMYKYLKNMVLNLFGSESLKKMLPEVEEVAKNKLKRWSGQTSVEMKEATAN
MIFDLTAKKLISYDSETSSENLRESFVAFIQGLISFPIDIPGTAYHKCLQGRKKAMKMLKT
MLEERRAKPRKEGTDFFDYVLEELQKNDIILTEAIALDLMFVLLFASFETTSLAITLATKFL
HDHPLALKELTEEHEAIIRSRENPASGLTWKEYKSMKFTFQVINETVRLANIVPAIFRKTL
TDINFKGYTIPAGWAVMVCPPAVHLNPAKYQDPLDFNPWRWEGVEINGATRNFMAFG
GGMRFCVGTDFTKVQMAVFLHSLVTKYRWQTIGGGNTVRTPGLQFPNGYHVRISEKD
EKIL

> *Digitalis purpurea* (DpCYP87A106)
MSLVAISIGAILIVIITNCVFKWRNRSLSSGGILPPGSFGWPLIGETLHFFTPNTSFDVTPF
VKDRMKRYGPIFKTSLVGVPVIVSTDAELNNFIFQQEGQTFQSWYPSTFTEIFGRENLS
TLHGFMYKYFKNMVLGLFGPESLKTMISEVENTSNINLKRWSANGTVELKDAIAEMIFE
LTAKKLISYELEKSPYNLRDNFVAFIDGLISFPLNIPGTAYYKCLQGRKNAIKMLRDMLHE
RREKPRETQTDFFDYVLEELQKQDTIITETLALDLMFVLLFASHETASIALTLAMKFLVDH
PLVLDKLTEEHDEIIKMREDPNSGLTWNEYKSMKFTFQFINETLRLANIAPLIFRKALTET
EFKGYTIPAGWAVMVCLPAVHLDPTKYKNPLEFNPWRWEGVDTSVGSKTFMAFGGG
MRLCIGADFTKVQMAVFLHCLVTKYKWKTIKGGDIVRCPGLKFPNGFHVQMSEREAN
QKACK

> *Calotropis procera* (CpCYP87A103)
MMFAAIFLTTFLFIIVSRWIYRWRNPSCNGILPPGSMGLPIIGESLAYFTPYFKDDIPLFV
RERVQKYGPLFRTSLVGQSVIVSTDPEVNYYIFQQEGNLFQCWYSESVLKVLGEQSM
AVQAGAFHKYLKNLCLSLVGPENLKETLMYEMDQNTIEHLQSWGTIGNLDAKDATAEL
VFKLAARKIINYDEKKSGKKLRDCYKAFMDGFISFPLYIPGTAFYACIQGRKKALKVIKEV
FNQRRGIGATEEKQKVFVDYILEEVDNKESFITEGIALDLVFLLLFASHETTSTAMTMAM
KFITESPAVLAELVREHEAILKNRENPESGITWKEYKGMTFTHMVINETVRIANIAPGIFR
KVMKEVEIKGYTIPAGWTVMVCPSTVHMNPDKYENPLSFDPWRWEGQELHSASKNF
MAFGGGMRLCVGADFAKLQMAIFLHHLVTKFRWTITHGGDTVRKPGLLFPNGLHVQIS
AIKAN

> *Arabidopsis thaliana* (AtCYP87A2)
MWALLIWVSLLLISITHWVYSWRNPKCRGKLPPGSMGFPLLGESIQFFKPNKTSDIPPFI
KERVKKYGPIFKTNLVGRPVIVSTDADLSYFVFNQEGRCFQSWYPDTFTHIFGKKNVG
SLHGFMYKYLKNMVLTLFGHDGLKKMLPQVEMTANKRLELWSNQDSVELKDATASMI
FDLTAKKLISHDPDKSSENLRANFVAFIQGLISFPFDIPGTAYHKCLQGRAKAMKMLRN
MLQERRENPRKNPSDFFDYVIEEIQKEGTILTEEIALDLMFVLLFASFETTSLALTLAIKFL
SDDPEVLKRLTEEHETILRNREDADSGLTWEEYKSMTYTFQFINETARLANIVPAIFRKA

LRDIKFKDYTIPAGWAVMVCPPAVHLNPEMYKDPLVFNPSRWEGSKVTNASKHFMAF
GGGMRFCVGTDFTKLQMAAFLHSLVTKYRWEEIKGGNITRTPGLQFPNGYHVKLHKK
RD

> *Nicotiana benthamiana* (NbCYP87A122)
MISVGMCIGAFLVLIIIHWVYNWRNPRCNGKLPPGSMGWPLLGETIPFFAPNTSSDIAPF
VKDRMQRYGPIFRTSVVGRPVIASTDPDLNYFIFQQEGQLFQSWYPDTFTEIFGKQNV
GSLHGFMYKYLKNMVLNLFGPESLKKMMPEVEEAAKNKLKRWSGQTSVEMKEATAN
MIFDLTAKKLISYDSENSSENLRESFVAFIQGLISFPIDIPGTVYHKCLQGRKKAMKMLKT
LLEERRAKPRKEQSDFFDYVLEELQRKDTILTEAIALDLMFVLLFASFETTSLAITLATKF
LHDHPLALKELTEEHEAIIRRRENPASGLTWKEYKSMKFTFQVINETVRLANIVPAIFRK
ALRDVNFKGYTIPAGWAIMVCPPAVHLNPAKYQDPLEFNPWRWEGVEMNGASRNFM
AFGGGMRFCVGTDFTKVQMAVFLHSLVTKYRWETIQGGDTLRTPGLQFPNGYHIRLS
EKDEKIQ

> *Erysimum cheiranthoides* (EcCYP87A2)
MWALFIWVSLLLISITHWVYSWRNPKCRGKLPPGSMGFPLLGETIQFFKPNTSSDIPPFI
KERVKKYGSIFKTNLVGRPVIVSTDADLSYFVFQQEGRCFQSWYPDTFTKIFGEKNVG
SLHGFMYKYLKSMVLTLFGHDGLKKMLPQVEMTANKRLELWSNQDSVELKDATASMI
FYLTAKKLISHDPDKSSENLRANFVAFIQGLISFPFDIPGTAYHKCLQGREKAMKMLRN
MLQERRKKPRKYPSDFFDYVILEIQKEGTILTEEIALDLMFVLLFASFETTSLAITLAIKFLS
DDPAVLKRLTEEHETILRNREDADSGLTWEEYKSMTYTFQFINETARLANIVPAIFRKAL
RDIKFKDYTIPAGWAVMVCPPAVHLNSETYKDPLVFNPSRWEGSKATNASKHFMAFG
GGMRFCVGTDFTKLQMAAFLHCLVTKYRWEEIKGGNILRTPGLQFPNGYHVRLHKKE
T

> *Erysimum cheiranthoides* (EcCYP87A126)
MSWALCIWVSLVVTGITTLVYKWRNPKCSGKLPPGSMGLPLLGETIQFFKPNLTSDIQP
FIKERTKKYGPIFKTSLVGKSIIVTTDPDFSYFVFQQEGQSFQSWYPDTFVEIFGKQNLG
ALHGIIYKYLKHMVLSLFGFESLKNMLPEIEQTACKKLDLWSTQKSIELKESTANLIFDLT
AKKLISHDEEKSSENLRDNYVAFIDGLISFPINLPGTAFYKCLKGRERVMSSLRNMLKER
RKNPRKVASDFFDYVIEELKKEGTMLTESIALDLMFVLLFASFETTSLAITVAIKMLSDHP
SVLKRLTEEHEAILRNRKDPNSGLTWEEYKSMTYTFQFMNETARLANIAPLICRKALKDI
QYKNYTIPANWPVMVVPPAIHLDPNNYEDPLVFNPSRWEGSEFTNASKKFMAFGGGM
RFCIGTDFSKLQTAVFLHSLLTKYSWEHISGGNMLRSPGLQFPNGYHVKINKKEI

> *Oryza sativa* (OsCYP87A6)
MQPYLQLASLRLATTIPLAPRLYDANLLAASGAAMASSMAYIALLCAALAAVVALLRWA
YRWSHPRSNGRLPPGSLGLPVIGETLQFFAPNPTCDLSPFVKERIKRYGSIFKTSVVGR
PVVVSADPEMNYYVFQQEGKLFESWYPDTFTEIFGRDNVGSLHGFMYKYLKTLVLRLY
GQENLKSVLLAETDAACRGSLASWASQPSVELKEGISTMIFDLTAKKLIGYDPSKPSQV
NLRKNFGAFICGLISFPLNIPGTAYHECMEGRKNAMKVLRGMMKERMAEPERPCEDFF
DHVIQELRREKPLLTETIALDLMFVLLFASFETTALALTIGVKLLTENPKVVDALREEHEAI

IRNRKDPNSGVTWAEYKSMTFTSQVIMEIVRLANIVPGIFRKALQDVEIKGYTIPAGWGI
MVCPPAVHLNPEIYEDPLAFNPWRWQGKPEITGGTKHFMAFGGGLRFCVGTDLSKVL
MATFIHSLVTKYSWRTVKGGNIVRTPGLSFPDGFHIQLFPKN

> *Sesamum indicum* (SiCYP87A113)
MFPAAFYIGALLIIAITNWLYHWRNPRCNGVLPPGSMGWPLIGETLQFFAPNTSSDIPPF
VKQRMQRYGPVFKTSLVGRPVIVSTDADLNYFIFQQEGQLFQSWYPDTFTEIFGRQNV
GSLHGFMYKYLKNMVLNLFGPESLKKMLPEVEQASNRNLEKWTSQSIVEVKEATAKMI
FELTAKKLISYDSEKSTENLRENFVAFIQGLISFPVDIPGTAYHKCLQGRKKAMKMLKNM
LQERRERPRKVRTDFFDYVLEELQREDTLLTEAIALDLMFVLLFASFETTSLALTLATKF
LVEHPLVLKELTEEHEAIIKRRENPDSGLTWSEYKSMRFTFQFINETVRLANIVPGIFRK
AMRETKFKGYTIPAGWAIMVCPPAVHLDPKKYRDPLNFNPWRWEGIDTNGASRNFMA
FGGGMRFCVGTDFTKVQMAVFLHCLVTKYKWKAIKGGDILRTPGLQFPNGFHVHVTE
KDK

> *Olea europaea* (OeCYP87A93)
MFPAIFCIAAILVINITHWLYKWWNPDCNGVLPPGSMGWPLLGETLHFFAPNTSSDIPP
FVKERIHRYGPIFKTSLVGRPVIVSTDADLNYFIFQQEGQLFESWYPSTFVEIFGRQNVG
SLHGFMYKYLKNMVLNLFGPESLKKMLPDVEQASNIYLKRWSSQTMTEMKESTSKMIF
DLTAKKLISYDVEKSSDNLRKNFKAFIKGLISFPLNIPGTAYHKCLQGRKKAMKMLKSML
QERRERPRKFQSDFFDYVLEELQRQDTILTESIGLDLMFVLLFASHETTSLALTLAMKFL
SDHPLVLKELTEEHEAIIKKREIPDSGLTWNEYKSMKFTFQFINETVRLANIVPGIFRKAL
KDIKFKGHTIPAGWAVMVCPPAVHLNPAKYSNPLDFNPWRWEGVDTNGASRNFMAF
GGGMRFCVGTDFTKVQMAIFLHCLVTKYKWQAIKGGDIVRTPGLQFPNGFHISLSEKN
AQK

> *Homo sapiens* CYP11A1
MLAKGLPPRSVLVKGCQTFLSAPREGLGRLRVPTGEGAGISTRSPRPFNEIPSPGDNG
WLNLYHFWRETGTHKVHLHHVQNFQKYGPIYREKLGNVESVYVIDPEDVALLFKSEGP
NPERFLIPPWVAYHQYYQRPIGVLLKKSAAWKKDRVALNQEVMAPEATKNFLPLLDAV
SRDFVSVLHRRIKKAGSGNYSGDISDDLFRFAFESITNVIFGERQGMLEEVVNPEAQRF
IDAIYQMFHTSVPMLNLPPDLFRLFRTKTWKDHVAAWDVIFSKADIYTQNFYWELRQK
GSVHHDYRGILYRLLGDSKMSFEDIKANVTEMLAGGVDTTSMTLQWHLYEMARNLKV
QDMLRAEVLAARHQAQGDMATMLQLVPLLKASIKETLRLHPISVTLQRYLVNDLVLRDY
MIPAKTLVQVAIYALGREPTFFFDPENFDPTRWLSKDKNITYFRNLGFGWGVRQCLGR
RIAELEMTIFLINMLENFRVEIQHLSDVGTTFNLILMPEKPISFTFWPFNQEATQQ
